# Is there an advantage of using genomic information to estimate gametic variances and improve recurrent selection in animal populations?

**DOI:** 10.1186/s12711-025-00953-7

**Published:** 2025-02-17

**Authors:** Jean-Michel Elsen, Jérôme Raoul, Hélène Gilbert

**Affiliations:** 1 GenPhySE, Université de Toulouse, INRAE, Castanet-Tolosan, France; 2 Institut de l’Elevage, Castanet-Tolosan, France

## Abstract

**Background:**

Gametic variances can be predicted from the outcomes of a genomic prediction for any genotyped individual. This is widely used in plant breeding, applying the utility criterion (UC). This paper aims to examine the conditions to use UC for recurrent selection in livestock. Here, the UC for a selection candidate is the linear combination of the expected value of the future progeny (half of the candidate’s breeding value) and its predicted gametic variance weighted by a coefficient $\theta$ to be optimized.

**Results:**

First, generalizing previous results, we derived analytically the ratio of the variance of the candidate’s gametic variance and that of half of the candidate’s breeding value. This ratio depends strongly on the number of quantitative trait loci (QTL) affecting the trait and, to a lesser extent, on the distribution of QTL allele frequencies: highly unbalanced frequencies and a limited number of QTL (< 10) favor higher values of the ratio. Then, changes in average breeding values and genetic variances when recurrent selection in a population of infinite size is applied were analytically derived and analyzed for selection up to 15 generations: in this ideal situation, after 5 to 10 generations (depending on $\theta$), the expected breeding values were higher with selection on UC and the genetic variance was always higher than with selection on estimated breeding values. To describe the potential of the UC in more general situations, simulations were applied to a population of 1000 males and 1000 females, with various selection rates, numbers and allele frequencies of QTL, and $\theta$. These simulations were performed assuming independent QTL with known positions and effects. The best values for $\theta$ (i.e. providing the best genetic progress) were generally lower than 1, limiting the weight on the gametic variance. As expected from the analytical derivations, the gain in genetic progress from using UC was greatest when there were few QTL and allele frequencies were unbalanced, but they barely exceeded 5%.

**Conclusions:**

We conclude that the key factor to choose selection on UC rather than on estimated breeding values is the ratio between the variance of the gametic standard deviations and the variance of the breeding values (GEBV), which should be carefully evaluated.

**Supplementary Information:**

The online version contains supplementary material available at 10.1186/s12711-025-00953-7.

## Background

For a given trait, the genetic variance originates from the allelic polymorphism at the genes [quantitative trait loci (QTL)] involved in the determinism of its phenotypes. For each QTL, some individuals are homozygous and some are heterozygous. An individual that is heterozygous for at least one QTL produces gametes with variable genetic contents. This variability results in a “gametic variance” (also called Mendelian sampling variance [1] or within family sampling variance [2]): the contribution of a parent to the breeding value of its progeny (in the sense of the sum of the effects of QTL genotypes on the trait of interest) varies according to the transmitted alleles. Individuals in a population have different genetic contents, and therefore differ in gametic variances.

Using phenotyped and genotyped individuals, effects of single nucleotide polymorphisms (SNPs) on a trait of interest can be predicted. It is then possible to decompose the genetic variability of a population into its elementary components and, therefore, to predict the gametic variances of the genotyped individuals. This opportunity can be exploited for the genetic improvement of populations. In the plant breeding literature, several approaches have been proposed in which breeders must both choose lines from an available panel and optimize mating plans to create F1 generations from which the next generation of pure lines are generated, such as recombinant inbred lines (RIL) or doubled haploids (DH). Most plant breeding approaches do not aim at long-term improvement of populations but at promoting the emergence of exceptional lines that can take a significant share of the seed market. The oldest approach, and probably the most used, is selection on the utility criterion (UC), i.e. prediction of the genetic value of the best progeny of an F1 cross, which must be distinguished from the average value of the progeny of that F1 candidate [3–6]. The UC linearly combines the expected value of the future progeny of the F1 cross to be produced (in practice the average of the genomic estimated breeding values (GEBV) of the parental lines) and the prediction of the gametic variance that this F1 may produce. Other criteria have been proposed, some of which aim at long-term improvement of the populations, i.e. the optimum haploid value, which is the maximum value of a theoretical individual that combines the most favorable QTL alleles or haplotypes that are carried by the F1 candidate [7], the expected maximum haploid breeding value, which predicts the value of the best progeny of the F1 candidate among a set of finite size [8], and the optimal population value, which is the value of the best progeny that would be produced by the population of selected individuals after an infinite number of generations [9].

Considering gametic variances for genetic improvement of livestock has been little studied. Producing future exceptional breeders has long been a goal in very competitive production sectors, in which the value of the best individuals can be very high (race horses and dairy cattle). However, progressive accumulation of genetic progress, i.e. improvement of the average breeding value of the population, through recurrent selection is most often the norm. Various criteria were compared in [10], including UC and the probability of finding an extreme individual in the offspring of a breeding candidate. This study assumed known GEBV and gametic variances of the candidates, and did not simulate genomes explicitly (positions and effects of SNPs). In addition, only one generation of selection was considered. A criterion equivalent to UC was evaluated in [11] by simulation of recurrent genomic selection for 10 generations, after a long historical phase to establish a realistic genomic structure and to construct a reference population. This study considered different hypotheses for the number of QTL (20 or 200), heritabilities and weight of the gametic variance in the UC. The results showed that the gain due to information on gametic variances was negligible (or even negative) with 20 QTL, but increased with the number of QTL, which is in contradiction with predictions from [4].

In this paper we propose to extend and to re-evaluate by simulation the results of [10, 11] regarding the properties of UC for recurrent selection for animal populations. First, we extended the algebraic elements developed by [4] to the case of outbred animal populations that are subject to recurrent purebred selection. Then, the conditions of success for the use of UC were explored and evaluated by computing the genetic gain at different time horizons, in a number of scenarios that differed by the initial conditions and by the type of selection performed. This study was carried out under the assumption of additive inheritance due to the segregation of independent QTL with known effects, so results should be considered to be optimistic as to the usefulness of UC for long-term improvement of livestock populations.

## Methods

### Base model: notations and assumptions

A population with discrete generations ${G}_{\tau }$ ($\tau =\text{0,1}\cdots T$) was simulated. Each generation included $N$ individuals of each sex, among which reproductive candidates, ${N}_{m}$ males and ${N}_{f}$ females, were genotyped for SNPs across the genome. Among these, ${S}_{m}$ males and ${S}_{f}$ females were selected and contributed to the next generation. The selection rates will be noted ${p}_{m}={S}_{m}/{N}_{m}$ and ${p}_{f}={S}_{f}/{N}_{f}$.

The trait was determined by $Q$ independent QTL that were in linkage equilibrium. The QTL had additive genetic effects (classical model $\left\{-{a}_{q},0,{a}_{q}\right\}$ for genotypes ${g}_{q}=\left\{{A}_{q}{A}_{q}, {A}_{q}{B}_{q}, {B}_{q}{B}_{q}\right\}$), without dominance or epistasis. The ${A}_{q}$ allele was systematically designated as the least frequent allele in the non-selected population. The allele effects (${a}_{q},q=1\cdots Q$), considered as perfectly known, were sampled from a normal distribution $N(0,1$), and then standardized to a genetic variance (i.e. the variance of the true breeding values (TBV)) of 1 in the first generation, before selection. The genotype ${XM}_{iq}$ of the ${i}^{th}$ male candidate at the ${q}^{th}$ QTL is coded $\left\{-\text{1,0},1\right\}$ and similarly for ${XF}_{jq}$ for the ${j}^{th}$ female candidate). The vectors ${{\varvec{X}}{\varvec{M}}}_{{\varvec{i}}}=({XM}_{i1},{XM}_{i2}\cdots {XM}_{iQ})$ and ${{\varvec{X}}{\varvec{F}}}_{{\varvec{j}}}$ gather the genotypes of, respectively, the ${i}^{th}$ male and the ${j}^{th}$ female candidate. Frequencies of the genotypes ${g}_{q}$ at generation ${G}_{\tau }$ are denoted by ${F}_{{g}_{q}\tau }$, the allele frequencies by ${f}_{{A}_{q}\tau }$ and ${f}_{{B}_{q}\tau }$. A beta distribution was used to simulate QTL allele frequencies, with parameters $\alpha =\beta$. In the following, we will show that the formula developed for very simple situations show a strong impact on the results of allelic frequencies.distribution To account for this, we considered several values for the parameters $\alpha \left(=\beta \right) , \text{ranging from }0.05\text{ to }1.0$. Additional file 1 Figure S1 provides details for this choice.

### Selection criterion

We are interested in the distribution of the gametic values that each of the candidates can produce. For a candidate ($i$) the mean of this distribution is $\frac{1}{2}{TBV}_{i}$ and its variance is denoted by ${ECT}_{i}^{2}$. The UC for candidate $i$ is then ${UC}_{i}{=\frac{1}{2}TBV}_{i}+\theta {ECT}_{i}.$

The $TBV$ for male *i* is obtained as ${TBV}_{i}=\sum_{q}{XM}_{iq}{a}_{q}$ and its gametic standard deviation is ${ECT}_{i}=\sqrt{\sum_{\left\{q |{XM}_{iq}=0 \right\}}{\sigma }_{q}^{2}}=\sqrt{\sum_{q}{\delta }_{iq}{\sigma }_{q}^{2}}$, with ${\delta }_{iq}=1$ if locus $q$ is heterozygous in individual $i$, and = *0* otherwise, *i.e.* ${\delta }_{iq}=1-{XM}_{iq}^{2}$. For a heterozygous locus $q$ in individual $i$, the gametic variance is ${\sigma }_{q}^{2}=\frac{{a}_{q}^{2}}{4}$.

While in plant breeding (e.g. [4]) ${UC}_{i}$ is a measure of the value of the ${i}^{th}$ possible cross between two inbred lines, here it is a measure of the value of the best gametes produced by a candidate. These two applications of ${UC}_{i}$ differ from each other on two levels. On the one hand, in the plant breeding scenario, parameter $\theta$ is equal to $i\left(p\right)$, i.e. the selection intensity corresponding to the selection rate $p$ that would be applied to future progeny of the cross; here, parameter $\theta$ is one of the variables to be optimized, with the objective of maximizing the expectated genetic progress in the ${T}^{th}$ generation. On the other hand, the quantity ${\frac{1}{2}TBV}_{i}$, which is the mean of the distribution of the gametic values produced by candidate $i$, replaces ${TBV}_{i}$ as the mean in the plant breeding context, which is the mean of the distribution of the genetic value of pure lines that can be produced from cross $i$.

The TBV of all individuals of the starting generation (${G}_{0}$) were normally distributed with mean ${\mu }_{g0}$ and variance ${\sigma }_{g0}^{2}$. The mean ${\mu }_{g\tau }$ of the distribution of the TBV of individuals of the ${G}_{\tau }$ generation was estimated as the average TBV of the $N$ sires of this generation. Results were obtained from $Nsim$ replicate simulations. Random samplings (to produce gametes and matings) were carried out using the NAg library [12].

Variances (and expectations) of a random variable $Y$ that is defined at two levels with subscripts $a$ and $b$ will be denoted, for instance, as ${var}_{b}\left({Y}_{ab}\right)$, indicating that the variance is within level $a$ and across levels of $b$. For instance, the variance of the TBV of the progeny (individuals indicated by subscript $k$) of sire $i$ is ${var}_{k}\left({TBV}_{ik}\right)={v}_{i}$, and this variance varies between sires.

### Conditions for the effectiveness of a selection based on the utility criterion

In the case of crosses between inbred lines, Zhong and Jannink [4] highlighted the role of the ratio ${{t}_{ZJ}=var}_{i}\left({ECT}_{i}\right)/{var}_{i}\left({TBV}_{i}\right)$ in the relative efficiency of selection on ${UC}_{i}{=TBV}_{i}+i(p) {ECT}_{i}$: the higher this ratio, the more the variance of the gametic standard deviation contributes to the total variance of UC, giving more importance to this information in the ranking of the possible crosses. If ${t}_{ZJ}=0$, the choice is made exclusively on a classical TBV criterion. The gametic variance of a cross between inbred lines is directly related to the number of heterozygous QTL in this cross. In the case of independence between QTL and equal QTL effects, Zhong and Jannink [4] proposed a simple equation (Eq. 11 in their paper) that explains this link: $${t}_{ZJ}=1-\frac{{0.5}^{2Q-1}}{Q}{\left[{\sum }_{{Q}_{m}=0}^{Q}\left(\begin{array}{c}Q\\ {Q}_{m}\end{array}\right)\sqrt{{Q}_{m}}\right]}^{2}$$, where ${Q}_{m}$, the number of heterozygous QTL among the $Q$ QTL, is a random variable assumed to follow a binomial distribution $\mathcal{B}(0.5, Q)$.

In our situation, with ${UC}_{i}{=\frac{1}{2}TBV}_{i}+\theta {ECT}_{i}$, a similar phenomenon occurs, with slightly different equations (the equations developed below are for males, with subscript $i$, but can also be applied to females with subscript $j$). Let us consider ${t=var}_{i}\left({ECT}_{i}\right)/{var}_{i}\left(\frac{1}{2}{TBV}_{i}\right)$ as an indicator of the relative efficiency of selection on ${UC}_{i}$.

Assuming independent QTL, ${var}_{i}\left({TBV}_{i}\right)={\sigma }_{g0}^{2}={var}_{i}\left(\sum_{q}{XM}_{iq}{a}_{q}\right)=\sum_{q}\left({f}_{{A}_{q}{A}_{q}}+{f}_{{B}_{q}{B}_{q}}-{\left({f}_{{B}_{q}{B}_{q}}-{f}_{{A}_{q}{A}_{q}}\right)}^{2}\right){a}_{q}^{2}$. Under Hardy–Weinberg equilibrium, this simplifies to: ${var}_{i}\left({TBV}_{i}\right)=\sum_{q}2{f}_{{A}_{q}}{f}_{{B}_{q}}{a}_{q}^{2}$.

With equal QTL effects (${a}_{q}=a \; \forall q$) ${var}_{i}\left({TBV}_{i}\right)=E\left({Q}_{m}\right){a}^{2}$. With the additional hypothesis that all QTL have the same allele frequencies (${f}_{{A}_{q}}={f}_{A} \; and \; {f}_{{B}_{q}}={f}_{B} \; \forall q$), ${var}_{i}\left({TBV}_{i}\right)=2{f}_{A}{f}_{B}Q{a}^{2}$. These are the conditions corresponding to the ${t}_{ZJ}$ ratio formula of [4] mentioned above, giving in their case ${var}_{i}\left({TBV}_{i}\right)=0.5Q{a}^{2}$.

The variance ${var}_{i}\left({ECT}_{i}\right)$ is obtained using ${var}_{i}\left({ECT}_{i}\right)={E}_{i}\left({ECT}_{i}^{2}\right)-{\left({E}_{i}\left({ECT}_{i}\right)\right)}^{2}$.

Denoting ${TBV}_{ik}$ as the TBV of progeny $k$ of sire $i$, and applying the usual equality ${var}_{X}\left(X\right)={E}_{Y}\left({var}_{X}\left(X[Y\right)\right)+{var}_{Y}\left({E}_{X}\left(X[Y\right)\right)$ to the variance of the TBV of all possible progeny from the ${G}_{0}$ generation (excluding selection, therefore without changes in genetic variance between generations), ${var}_{ik}\left({TBV}_{ik}\right)={\sigma }_{g0}^{2}={E}_{i}\left({ECT}_{i}^{2}+\frac{1}{2}{\sigma }_{g0}^{2}\right)+{var}_{i}\left(\frac{1}{2}{TBV}_{i}\right)={E}_{i}\left({ECT}_{i}^{2}\right)+\frac{3}{4}{\sigma }_{g0}^{2}$ and, therefore, ${E}_{i}\left({ECT}_{i}^{2}\right)=\frac{1}{4}{var}_{i}\left({TBV}_{i}\right)$.

$$E_{i} \left( {ECT_{i} } \right) = E_{i} \left( {\sqrt {\mathop \sum \limits_{q} \delta_{iq} \frac{{a_{q}^{2} }}{4}{ }} } \right) = \mathop \sum \limits_{{\left\{ {XM_{i} } \right\}}} prob\left( {XM_{i} } \right)\sqrt {\mathop \sum \limits_{q} \delta_{iq} \frac{{a_{q}^{2} }}{4}{ }} .$$

Under the same simplifying assumptions (equality of QTL effects and the same allele frequencies), $${E}_{i}\left({ECT}_{i}\right)={\sum }_{{Q}_{m}=0}^{Q}prob({Q}_{m})\sqrt{{Q}_{m}}\frac{a}{2}=\left({\sum }_{{Q}_{m}=0}^{Q}\left(\begin{array}{c}Q\\ {Q}_{m}\end{array}\right){\left(2{f}_{A}{f}_{B}\right)}^{{Q}_{m}}{\left(1-2{f}_{A}{f}_{B}\right)}^{{Q-Q}_{m}}\sqrt{{Q}_{m} }\right)\frac{a}{2}$$.

Finally, the ratio $t=1-4\frac{{\left({E}_{i}\left({ECT}_{i}\right)\right)}^{2}}{{var}_{i}\left({TBV}_{i}\right)}$ can then be written as:

1 $$t = 1 - \frac{{\left( {\mathop \sum \nolimits_{{Q_{m} = 0}}^{Q} \left( {\begin{array}{*{20}c} Q \\ {Q_{m} } \\ \end{array} } \right)\left( {2f_{A} f_{B} } \right)^{{Q_{m} }} \left( {1 - 2f_{A} f_{B} } \right)^{{Q - Q_{m} }} \sqrt {Q_{m} } } \right)^{2} }}{{2f_{A} f_{B} Q}}.$$

If ${f}_{A}={f}_{B}=0.5$, the Eq. (11) of Zhong and Jannink [4] results.

**Fig. 1:**
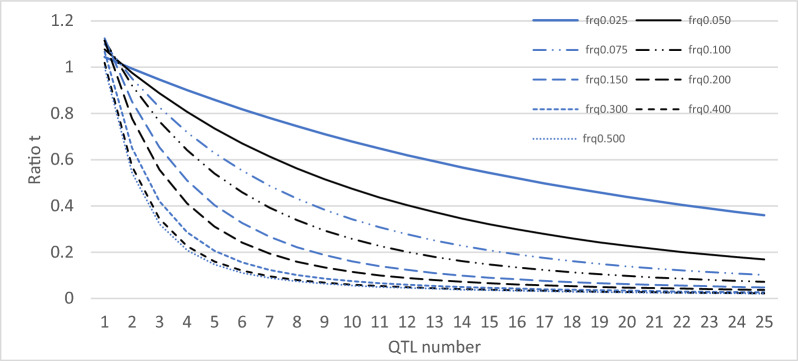
Ratio $t$ as a function of the number of QTL and their allele frequencies [Eq. (1)]. The ratio between the between reproducers variances of the standard deviation of gamete genetic values for a quantitative trait and of expected breeding values of their progeny depends on the number of QTL controlling the trait and QTL allele frequencies (QTL are assumed biallelic and the frequencies are the minor allele frequencies)

Figure 1 shows that the $t$ ratio decreased rapidly as the number of QTL increased, a result already described by Zhong and Jannink [4], and shows that more balanced the allelic frequencies faster this decrease.

With selection, the frequencies of unfavorable alleles decrease. Thus, we expect that the contribution of information on gametic variances is more important when the population has been selected for a long period.

### Algebraic expression of the effectiveness of recurring selection on the utility criterion

First, we examine the changes in the distribution of genetic values in the case of an infinitesimal model when selection is on the UC. To simplify the analysis, we considered the case where only males are selected (${S}_{f}={N}_{f}$) and assumed that the $TBV$ have a Gaussian distribution, which is questionable when the number of QTL is small.

In the short term, compared to a classical selection of males on their $TBV$ alone, selection on ${UC}_{i}{=\frac{1}{2}TBV}_{i}+\theta {ECT}_{i}$ results in a ${G}_{1}$ generation with a distribution of $TBV$ with a smaller mean and a greater variance. Additional file 2 Text S1 describes the details of the derivations.

Let ${\mu }_{\sigma 0}={E}_{i}\left[{ECT}_{i}\right]$ be the mean gametic standard deviation in generation ${G}_{0}.$ The mean of the genetic values in ${G}_{1}$ is ${\mu }_{g1}={E}_{ik}\left[{TBV}_{ik}|{UC}_{i}>{s}_{m}\right]={\mu }_{g0}+i({p}_{m})\frac{{cov}_{ik}({UC}_{i},{TBV}_{ik})}{{var}_{i}({UC}_{i})}\sqrt{v{ar}_{i}({UC}_{i})}$, with ${s}_{m}$ the selection threshold for UC that corresponds to the selected proportion ${p}_{m}$.

Assuming that ${TBV}_{i}$ and ${ECT}_{i}$ are independent, the above formula reduces to ${\mu }_{g1}={\mu }_{g0}+\frac{1}{2}i({p}_{m}){\sigma }_{g}\frac{1}{\sqrt{1+{\theta }^{2}{t}_{0}}}$, with ${t}_{0}={var}_{i}\left({ECT}_{i}\right)/{var}_{i}\left({\frac{1}{2}TBV}_{i}\right)$, i.e. the $t$ ratio in generation ${G}_{0}$.

The variance of the distribution of $TBV$ in the ${G}_{1}$ progeny is:

$$\begin{aligned} \sigma_{g1}^{2} & = \sigma_{TBV1}^{2} = var_{ik} \left[ {TBV_{ik} \left| {UC_{i} } \right. > s_{m} } \right] \hfill \\ & = E_{i} \left[ {var_{k} \left[ {TBV_{ik} \left| {i;UC_{i} } \right. > s_{m} } \right]} \right] + var_{i} \left[ {E_{k} \left[ {TBV_{ik} \left| {i;UC_{i} } \right. > s_{m} } \right]} \right], \hfill \\ \end{aligned}$$

which gives $${\sigma }_{g1}^{2}={\sigma }_{g0}^{2}\left(\frac{3}{4}+\frac{1}{4}{t}_{0}\right)-\frac{1}{4}{\sigma }_{g0}^{2}i({p}_{m})\left(i({p}_{m})-s\right)\frac{1+{\theta }^{2}{t}_{0}^{2}}{1+{\theta }^{2}{t}_{0}}+{\left({\mu }_{\sigma 0}+i({p}_{m})\frac{1}{2}{\sigma }_{g0}\frac{\theta {t}_{0}}{\sqrt{1+{\theta }^{2}{t}_{0}}}\right)}^{2}$$.

As demonstrated in Additional file 2 Text S1, following the hypothesis made by [13] of no change in gametic variances under selection, the ratio of variances in ${G}_{1}$ is ${t}_{1}={t}_{0}\frac{{\sigma }_{g0}^{2}}{{\sigma }_{g1}^{2}}$ and the invariable distribution of ${ECT}_{i}$ has mean ${\mu }_{\sigma 0}={\mu }_{\sigma }=\sqrt{\frac{1}{4}{\sigma }_{{g}_{0}}^{2}(1-{t}_{0})}$ and variance ${{v}_{\sigma }=var}_{i}\left[{\sigma }_{i0}\right]=\frac{1}{4 }{{\sigma }_{g0}^{2} t}_{0}$. We also show that ${v}_{\sigma }+{\mu }_{\sigma }^{2}={E}_{i}\left[{ECT}_{i}^{2}\right]$ is the variance of the Mendelian sampling for the paternal gametes.

The above formulas to obtain the moments of the distributions in ${G}_{1}$ from those of ${G}_{0}$ can be applied recursively, i.e.:

$$\mu_{g\tau + 1} = \mu_{g\tau } + \frac{1}{2}i\left( {p_{m} } \right)\sigma_{g\tau } \frac{1}{{\sqrt {1 + \theta^{2} t_{\tau } } }},$$

$$\sigma_{g\tau + 1}^{2} = \sigma_{g\tau }^{2} \left( {\frac{3}{4} + \frac{1}{4}t_{\tau } } \right) - \frac{1}{4}\sigma_{g\tau }^{2} i\left( {p_{m} } \right)\left( {i\left( {p_{m} } \right) - s} \right)\frac{{1 + \theta^{2} t_{\tau }^{2} }}{{1 + \theta^{2} t_{\tau } }} + \left( {m_{\sigma } + i\left( {p_{m} } \right)\frac{1}{2}\sigma_{g\tau } \frac{{\theta t_{\tau } }}{{\sqrt {1 + \theta^{2} t_{\tau } } }}} \right)^{2} ,$$

$$t_{\tau } = 4\frac{{v_{\sigma } }}{{\sigma_{g\tau }^{2} }} = t_{0} \frac{{\sigma_{g0}^{2} }}{{\sigma_{g\tau }^{2} }},$$

$$m_{\sigma } = E_{i} \left[ {\sigma_{i0} } \right] = \frac{1}{2}\sigma_{g0} \sqrt {1 - t_{0} } ,$$

$$v_{\sigma } = var_{i} \left[ {\sigma_{i0} } \right] = \frac{1}{4 }\sigma_{g0}^{2} t_{0} .$$

**Fig. 2:**
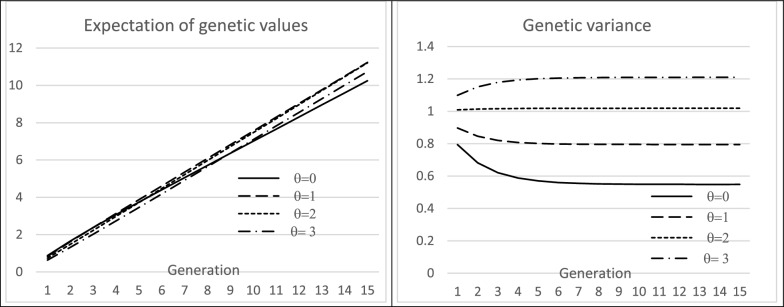
Mean genetic values and genetic variance after $T$ generations of selection as a function of the coefficient θ (initial ratio ${t}_{0}$ = 0.1, selection rate 10%) in Utility Criterion. Individuals are selected on the Utility Criteria defined by ${UC}_{i}{=\frac{1}{2}TBV}_{i}+\theta {ECT}_{i}$ (${TBV}_{i}$ is the true breeding value and ${ECT}_{i}$ the gametic standard deviation of individual $i$). A deterministic algebraic model iteratively describes the evolution of the mean genetic values and variances. An infinitesimal model with a Gaussian distribution of QTL effects is assumed

As an example, Fig. 2 shows, under the assumption made to obtain those formulas, the values of ${\mu }_{g\tau }$ and ${\sigma }_{g\tau }^{2}$ as a function of the $\theta$ coefficient of the UC, for a constant selection rate of 10% and an initial variance ratio ${t}_{0}=$ 0.1. Selection on UC turned out to be more profitable as the number of generations increased. In the long term, the genetic variances stabilized (as the equations correspond to the case of a population of infinite size), with increasing values as $\theta$ increases, allowing greater genetic gain in the long term. The limit of ${\sigma }_{gT}^{2}$ for $\theta =0$ was $1/(1+i({p}_{m})\left(i\left({p}_{m}\right)-{s}_{m}\right))$. It is notable that, for high values of $\theta$, the genetic variance increased during the first generations.

**Fig. 3:**
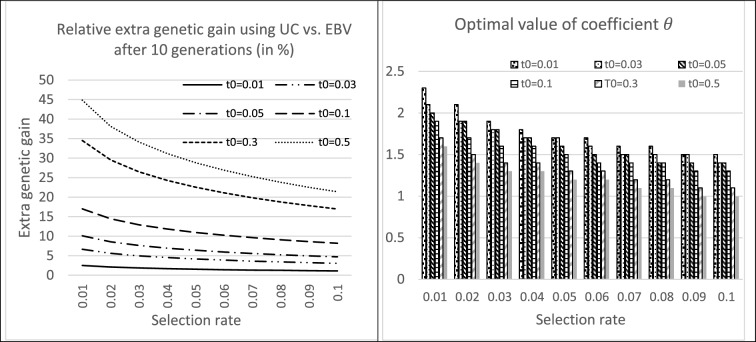
Relative efficiency of Utility Criterion selection over 10 generations as a function of selection rate ${p}_{m}$ and the initial ratio ${t}_{0}$. Individuals are selected either on the Utility Criteria defined by ${UC}_{i}{=\frac{1}{2}TBV}_{i}+\theta {ECT}_{i}$ (${TBV}_{i}$ is the true breeding value and ${ECT}_{i}$ the gametic standard deviation of individual $i$) or on their $TBV.$ A deterministic algebraic model iteratively describes the evolution of the mean genetic values and variances. An infinitesimal model with a Gaussian distribution of QTL effects is assumed and generations are non-overlapping. The maximum relative gain and the corresponding optimal values of $\theta$ depends on the selection rate (assumed identical in both sexes) and the initial ${t}_{0}$ ratio between the between reproducers variances of the standard deviation of gamete genetic values and of the expected breeding values of their progeny

With the example of a selection over 10 generations (results were similar for other durations), it clearly appeared (Fig. 3) that the effectiveness of using the gametic variance to choose breeders was better when the ratio ${t}_{0}$ was high and the selection rate ${p}_{m}$ was low. The optimal value of θ decreased with selection intensity, i.e. use of the gametic variance in selection was more useful as the selection rate ${p}_{m}$ decreased i.e. when the selection intensity is higher. In addition, the optimal $\theta$ values (i.e.; giving the highest genetic gains after 10 generations) were lower when ${t}_{0}$ was high, suggesting the existence of an optimum for the use of gametic variance.

It is important to note that this simple model assumes infinite numbers of breeders and of QTL, and therefore does not describe the loss of genetic variability due to drift. More detailed models that account for changes in the number of segregating QTL, and as a consequence, of the distribution of the gametic variances have not been developed yet.

### Heredity of gametic variances

The previous model assumes that the distribution of Mendelian sampling terms is not impacted by selection. In their discussion, Bijma et al. [10], who used the same hypothesis, noted that heterozygosity at a locus $q$ is transmitted, since regardless of the allele frequencies ${f}_{{A}_{q}}$ and ${f}_{{B}_{q}}$, a heterozygous individual ${A}_{q}{B}_{q}$ has a 50% chance of having heterozygous progeny, which is a greater probability than the average over all genotypes $(2{f}_{{A}_{q}}{f}_{{B}_{q}})$ when ${f}_{{A}_{q}}{\ne f}_{{B}_{q}}$. Considering the case of a single QTL with frequency $f$ for the minor allele, Bijma et al. [10] proposes a formula for the “heritability” of heterozygosity based on parent–offspring regression: ${h}_{het}^{2}=\frac{1-4f(1-f)}{1-2f(1-f)}$, demonstrating that this heritability is higher when MAF is smaller.

This generalizes to the heritability of heterozygosity involving *Q* QTL. In the simple case when QTL are unlinked and in linkage equilibrium and the QTL effects and allele frequencies are the same for all QTL (${f}_{{A}_{q}}=f$, $\forall q$), we find (see Additional file 3 Text S2) that the above formula of Bijma et al. [10] for a single locus is still valid. When allele frequencies vary between QTL, the correlation between the level of heterozygosity of parents (${Q}_{mi}$) and progeny (${Q}_{mk}$) is:

$$r\left( {Q_{mi} ,Q_{mk} } \right) = \frac{1}{2}\frac{{\mathop \sum \nolimits_{q = 1}^{Q} fh_{q} \left( {1 - 2fh_{q} } \right)}}{{\mathop \sum \nolimits_{q = 1}^{Q} fh_{q} \left( {1 - fh_{q} } \right)}},$$

where ${fh}_{q}$ is the frequency of heterozygotes at locus $q$. This same result was obtained in a quite different way by [14], with a generalization to multiallelic loci by [15].

In more general situations, correlations between the gametic standard deviations of parents and offspring can be obtained by simulations, which is what was used here, using the procedures described in the “basic model” section, with $T=2$ generations and $N={N}_{m}={N}_{f}=1000$ individuals of each sex per generation, all being retained as breeders. Various values of the parameters $\alpha =\beta$, from 0.2 to 1.0, and of the number of QTL, from 5 to 1000, were simulated $Nsim=1000$ times, resulting in allele frequencies that varie between QTL and between simulations. The effects of all QTL were assumed to be equal and set to $\left|{a}_{q}\right|=2/\sqrt{Q} \forall q$, such that gametic variances could be standardized. For individual $i$, the gametic variance ${ECT}_{i}^{2}$ is then proportional to the number ${Q}_{mi}$ of its QTL that are in the heterozygous state ${(ECT}_{i}^{2}=\sum_{\left\{q |{XM}_{iq}=0 \right\}}\frac{{a}_{q}^{2}}{4}={Q}_{mi}\frac{{\left(2/\sqrt{Q}\right)}^{2}}{4}=\frac{{Q}_{mi}}{Q}$).

**Fig. 4:**
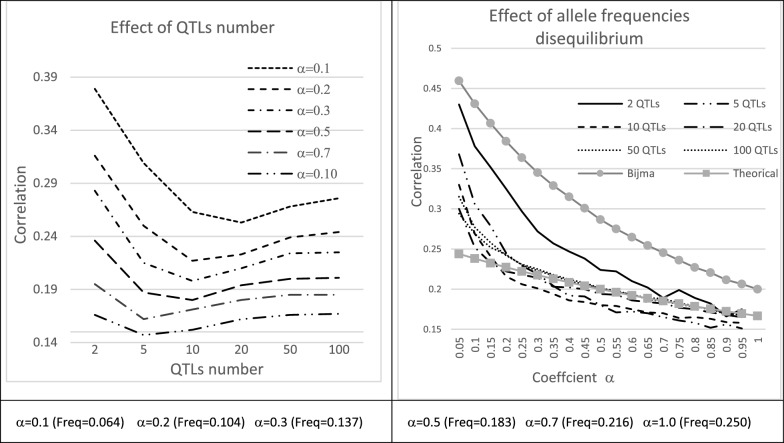
Simulated correlations between the gametic standard deviations of parents and offspring according to the number of QTL and the distribution of QTL allele frequencies. Simulations (1000 replicates / case) were performed for various numbers of QTL (*Q* = 2 to 1000) and distributions of QTL allele frequencies (defined by parameters of a Beta($\alpha ,\alpha$) distribution). The population consisted of $N={N}_{m}={N}_{f}=1000$ individuals of each sex per generation. The effects of the QTL were assumed to be equal and set to $\left|{a}_{q}\right|=2/\sqrt{Q} \forall q$

As expected, these correlations between the gametic standard deviations of parents and offspring (Fig. 4) were higher when the QTL allele frequencies were unbalanced. The correlations were also influenced by the number of QTL, with a U-shaped curve, with the minimum being reached for a number of QTL that was greater when the average minor allele frequency (${A}_{q}$) was low. The curve tracing the evolution of the heritability proposed by [10] as a function of the frequency $f$ was found above the simulated values. The “theoretical” expectation curve of $r\left({Q}_{mi},{Q}_{mk}\right)$ (estimated with the assumptions of the simulations, see Additional file 3 Text S2) properly described the impact of the distribution of the frequency of the ${A}_{q}$ allele on the correlation when the number of QTL was relatively large and the allele frequencies were not extreme, but underestimated the correlation for low values of $\alpha$.

**Fig. 5:**
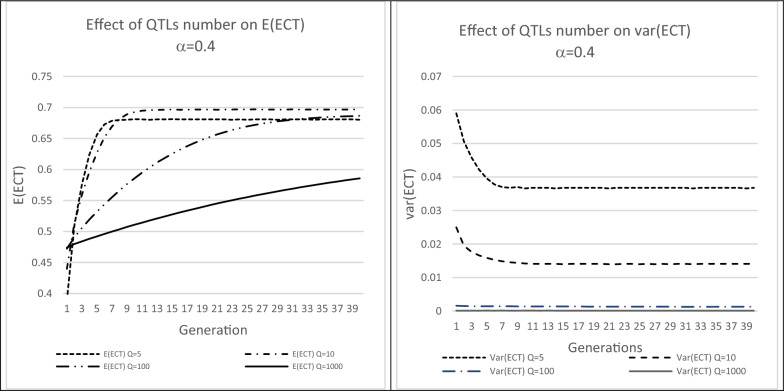
Changes in the mean and variance of gametic standard deviations (ECT) under selection on the Utility Criterion. Selection of 50 sires among 1000 on ECT, with a Beta distribution of coefficients $\alpha$ = $\beta$  = 0.4, corresponding to an average minor allele frequency of 0.159. Females were not selected. Each case was replicated 1000 times

Due to its inheritance, the gametic variance will change under selection on a criterion using it, such as UC. This is reinforced when the selection criterion is the gametic standard deviation itself (${UC}_{i}{=ECT}_{i}$). Figure 5 reports changes in the mean and variance of ECT with selection on ECT of 50 sires among 1000, *i.e.* with a Beta distribution of coefficients $\alpha$ = $\beta$  = 0.4 that corresponds to an average frequency of the ${A}_{q}$ allele of 0.159 (figure S1).

As shown on Fig. 5, the mean ECT increased under selection and quickly reached a plateau when the number of QTL was small (5 to 10). Conversely, the variance of the gametic standard deviations decreased over time and stabilized after around ten generations. For 100 or 1000 QTL, this variance was very low.

Selection based on ECT favors the most heterozygous individuals. Following [16], it is expected that an equilibrium will be reached, where all allele frequencies are equal to 0.5. At equilibrium, following the algebra of [4], we find that $$E\left({ECT}_{i}\right)=\frac{2}{\sqrt{Q}}{\sum }_{{Q}_{m}=0}^{Q}\left(\begin{array}{c}Q\\ {Q}_{m}\end{array}\right)\sqrt{{Q}_{m}}$$ and $$var\left({ECT}_{i}\right)=\frac{4}{Q}{\sum }_{{Q}_{m}=0}^{Q}\left(\begin{array}{c}Q\\ {Q}_{m}\end{array}\right){Q}_{m}-{E\left({ECT}_{i}\right)}^{2}$$. The observed plateaus (Fig. 5) corresponded perfectly to these theoretical moments for $Q$ = 5 (0.680 and 0.0368) and for $Q$ = 10 (0.696 and 0.014).

### Effectiveness of a recurring selection on a utility criterion

#### Approach

The algebraic formulas derived above assumed invariance of the distribution of gametic standard deviations (${ECT}_{i}$). It showed that recurrent selection on UC (${UC}_{i}{=\frac{1}{2}TBV}_{i}+\theta {ECT}_{i}$) can in the long term be more effective than a selection on TBV (or on GEBV in practice). The short-term increase in the variance of gametic standard deviations described in the previous simulations suggested an even higher efficiency of UC as compared to selection on TBV. These extra gains depend on the number of QTL for the trait under selection and QTL allele frequencies: fewer QTL and more unbalanced frequencies results in higher gain. The formulas assumed an infinite population size, thus neglecting the reduction in genetic variance due to drift and, therefore, overestimating long-term progress.

We now evaluate through simulations different scenarios of recurrent selection on UC (see the “basic model” section for a description of the simulations). The 1980 cases studied are described in Table 1. In all cases, reproducers (${S}_{m}$ males and ${S}_{f}$ females) were chosen amongst $N={N}_{m}={N}_{f}=1000$ individuals. Each case was simulated $Nsim$ =1000 times.

**Table 1:** Summary of the simulated scenarios for selection based on a Utility Criterion (UC)

Parameter	Abbreviation	Values
Number of selected males	${S}_{m}$	5, 10, 50
Number of selected females	${S}_{f}$	50, 500, 1000
Coefficient for the Beta distribution of alleles frequencies	$\alpha$	0.2, 0.4, 0.6, 0.8,1.0
Number of QTL	$Q$	5, 10, 100, 1000
Coefficient for UC computation	$\theta$	0.0, 0.1, 0.2…0.9, 1.0

For each replicate, each selection criterion (defined by the value of the $\theta$ coefficient) and each condition (defined by the numbers of individuals retained, ${S}_{m}$ and ${S}_{f}$, the number $Q$ of QTL, and coefficient $\alpha$ of the distribution of allele frequencies), the genetic gain was estimated as the mean of the TBV of the $N$ = 1000 new males at generations ${G}_{2}, {G}_{5},$ and ${G}_{10}$: ${GG}_{\tau }=\frac{1}{N}{\sum }_{i=1}^{1000}{TBV}_{i(\tau )}$. The discounted cumulative gain was also computed, as ${DCGG}_{10}={\sum }_{\tau =1}^{10}{(\frac{1}{1+act})}^{\tau }{GG}_{\tau }$, where *act* is the discount rate, which was set to $act$ =0.1. The high rate of 0.1 accounts for the fact that generations actually cover several years.

For the different scenarios, classical selection (on ${TBV}_{i}$) was compared to the best UC (${UC}_{i}$) among the 10 evaluated values of $\theta$, using the mean values of ${GG}_{\tau } (\tau =\text{2,5} \; \text{or} \; 10)$ and ${DCGG}_{10}$ of the corresponding set of replicates. The best UC is the one which that maximized ${GG}_{\tau }$ $(\tau =\text{2,5} \; \text{or}\; 10)$ or ${DCGG}_{10}$.

## Results

An extensive presentation of the results is provided in Additional file 4 Tables S1 to S4. For clarity, only the scenarios with intense selection of males (${S}_{m}=5$) without selection of females (${S}_{f}=1000$) is reported here, as the trends were similar in all the other scenarios.

**Fig. 6:**
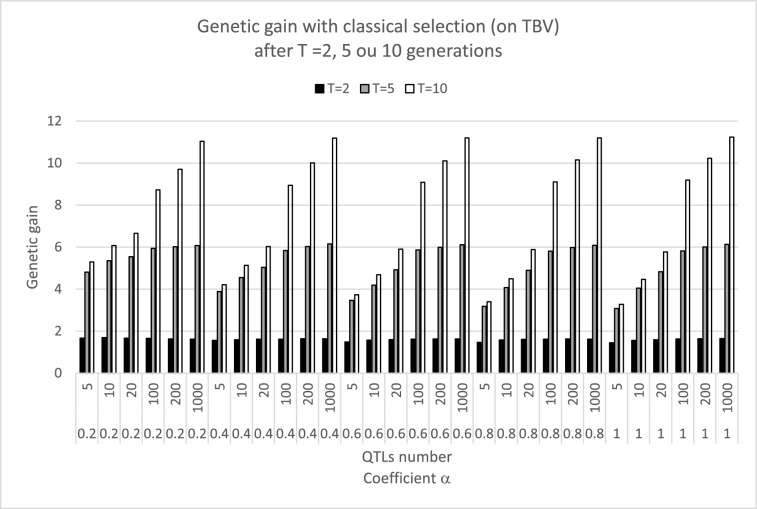
Genetic gain after 2, 5. and 10 generations for classical selection ($\theta$ = 0). Selection of ${S}_{m}=5$ sires among 1000 on their true breeding value. Females were not selected (${S}_{f}=1000$). Each case was simulated $Nsim$ =1000 times. Various numbers of QTL (5 to 1000) and distributions of alleles frequencies (defined by parameters of a Beta($\alpha ,\alpha$) distribution) were considered

The genetic progress obtained with classical selection followed expected trends (Fig. 6): higher gains were obtained with greater numbers of QTL. Low MAF (corresponding to low values of *α*) only affected scenarios with small numbers of QTL ($Q$ = 5 or 10): when few genes control a trait, each strongly contributes to the mean TBV and selection can fix favorable alleles that initially had a low frequency, significantly modifying the genetic mean.

Table 2 reports the observed values of the ratio ${{t}_{0}=var}_{i}\left({ECT}_{i}\right)/{var}_{i}\left(\frac{1}{2}{TBV}_{i}\right)$ according to parameters $Q$ and $\alpha$. In their considered ranges of variations, we see a very strong impact of the number of QTL and a more moderate influence of the difference between allele frequencies, which gets weaker as the number of QTL increases.

**Table 2:** Effects of the number of QTL and the distribution of minor allele frequencies (α) on the ${t}_{0}$ ratio

Number of QTL	α				
	0.2	0.4	0.6	0.8	1.0
5	0.565	0.429	0.363	0.321	0.29
10	0.371	0.25	0.194	0.167	0.152
100	0.035	0.022	0.017	0.015	0.014
1000	0.003	0.002	0.002	0.001	0.001

**Fig. 7:**
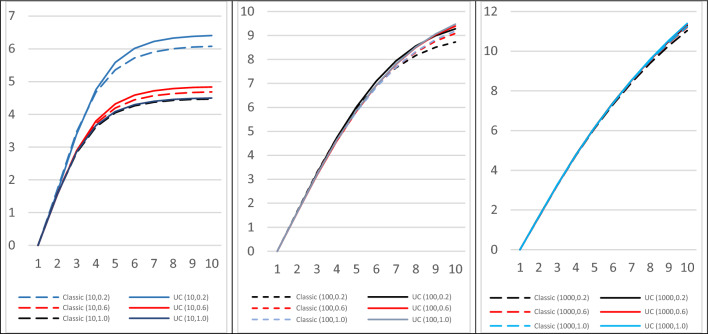
Changes of genetic mean for classical selection on true breeding values (${TBV}_{i}$) and on the most efficient utility criterion (${UC}_{i}$). Selection on ${TBV}_{i}$ or ${UC}_{i}{=\frac{1}{2}TBV}_{i}+\theta {ECT}_{i}$. Females were not selected (${S}_{f}=1000$). Number of QTL $Q=10, 100, 1000$, and coefficient $\alpha$ of the allele frequencies Beta distribution $\alpha =0.2, 0.6, 1.0$. Each case was simulated $Nsim$ =1000 times

The influence of the number of QTL and of the $\alpha$ parameter of the distribution of QTL allele frequencies on the changes in mean genetic values is illustrated in Fig. 7 for the case of ${S}_{m}=5, {S}_{f}=1000$. Taking gametic variances into account only provided extra genetic gain after at least four generations and was more visible when the number of QTL was small.

**Fig. 8:**
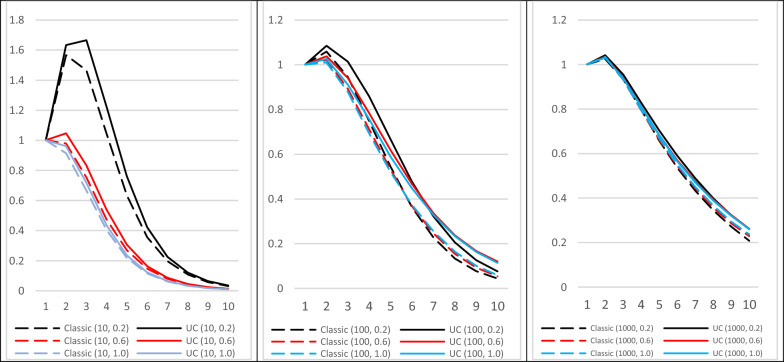
Changes of genetic variance for classical selection on true breeding values (${TBV}_{i}$) and the most efficient utility criterion (${UC}_{i}$). Selection of ${S}_{m}=5$ sires among 1000 on ${TBV}_{i}$ or${UC}_{i}{=\frac{1}{2}TBV}_{i}+\theta {ECT}_{i}$. Females were not selected (${S}_{f}=1000$). Number of QTL$Q=10, 100, 1000$, and coefficient $\alpha$ of the of allele frequencies Beta distribution$\alpha =0.2, 0.6, 1.0$. Each case was simulated $Nsim$ =1000 times

These changes over generations in the mean genetic values agreed with those of the genetic variances (Fig. 8). Excluding the second generation, variances gradually decreased, along similar trajectories, but less rapidly as the number of QTL increased. The increase observed for the second generation came from an increase in frequencies ${f}_{{B}_{q}2}$ of favorable but unfrequent alleles (${a}_{q}>0$) (for a single locus, the variance would be maximum when the allele frequencies are equal ${f}_{A\tau }={f}_{B\tau }$, and therefore variance would first increase if ${f}_{B0}<0.5$). This effect was exacerbated in the case with 10 QTL and strongly imbalanced allele frequencies ($\alpha$ = 0.2).

Additional file 4 Tables S1 and S2 report the additional gain when using UC instead of selection on TBV after five generations and additional cumulative gain over 10 generations for all simulated cases. The additional gain was very low for highly polygenic cases ($$Q$$ = 1000) (always lower than 2% for the cumulative gain) and even lower when the distribution across loci of allele frequencies was more uniform (high $$\alpha$$). The additional gain could exceed 5% when there were few QTL and the allele frequencies were more unbalanced, i.e. mean MAF lower. However additional gains were lower with five QTL compared with an intermediate number of QTL (10 or 100): with very few QTL, the favorable alleles are quickly fixed, the gametic variances becoming zero and, therefore, no longer provide any benefit. Although significant noise arose from the limited number of replications (1000), we can note that the additional gain was greater for small numbers of males ($${S}_{m}$$) and females ($${S}_{f})$$ retained as breeders, probably linked to greater opportunities for within-family selection by valuing the differences in gametic variances between families.

The optimal values of the UC coefficients $\theta$ are in Additional file 4 Tables S3 and S4. With few exceptions, they were always less than 1, showing that the range of tested values was sufficient. These low values for the optimal $\theta$ indicate that the weight given to gametic standard deviations in the choice of breeders was limited. This weight is given by ${var}_{i}\left({\theta ECT}_{i}\right)/\left[{var}_{i}\left(\frac{1}{2}{TBV}_{i}\right)+{var}_{i}\left({\theta ECT}_{i}\right)\right]={\theta }^{2}{t}_{0}/\left[1+{\theta }^{2}{t}_{0}\right].$ In the case of 10 QTL, which favorable to UC-based selection, the optimal $\theta$ was almost always 0.5, and the initial ${t}_{0}$ ratio was 0.37, 0.25, 0.19, 0.17, and 0.15 for, respectively, $\alpha$ equal to 0.2, 0.4, 0.6, 0.8, and 1.0 (see Table 2). Therefore, the weight given to the genetic standard deviations in the UC was 8.5, 5.9, 4.6, 4.0, and 3.7%, respectively.

## Discussion

### ***Influence of the ratio***${{\varvec{t}}}_{0}$***on the relative efficiency of the utility criteria***

Overall, our results show that taking gametic variances into account to select breeders results in greater rates of improvement of average genetic values. This phenomenon is caused by better exploitation of the genetic variation for the trait, which is less reduced, and therefore longer available for selection. However, the additional gain from UC selection (compared to a usual selection method based solely on estimated genetic values) is limited and almost negligible for highly polygenic traits, especially when the starting mean minor allele frequencies are higher.

Our report of greater genetic gaisn for selection on UC when the number of QTL is small is in agreement with [4]. The ratio ${{t}_{0}=var}_{i}\left({ECT}_{i}\right)/{var}_{i}\left(\frac{1}{2}{TBV}_{i}\right)$ of the variance of the gametic standard deviations and the breeding values variance, which decreases when the number of QTL and their minor allele frequencies increase (Table 2), is therefore a good predictor of the potential benefit of selection on UC (Fig. 7).

These observations contradict those of [11], who obtained a higher relative efficiency of UC with 200 QTL than with 20 QTL. Several causes can explain this difference: (1) they did not assume known QTL effects but estimated them using a genotyped and phenotyped reference population, for a trait with heritabilities ranging from 0.1 to 0.5; their results showed a tendency of a decrease in extra genetic gain provided by UC as compared to classical selection when heritability increased. In our case, the heritability is 1, for which this extra gain is certainly further reduced; (2) we present the efficiency of UC for the optimized values of $\theta$, which decreased with the number of QTL, while they only considered three values for this parameter (defined in their work as the future section intensity in the progeny generation); (3) they carried out only four replications per scenario, while we had 1000, and the results greatly varied between replicates: for example, the empirical distribution (based on 1000 simulations) of the cumulative genetic gain for the classical criterion ($\theta$ = 0) at $T$ = 5 generations for the case ${S}_{m}$ = 5, ${S}_{f}$ = 1000, $Q$ = 10, and $\alpha$ = 0.2 had a mean of 4.8 and a variance of 9.6 initial genetic standard deviations, and these values were 5.05 and 10.9, respectively, for the best UC ($\theta$ = 0.5); and (4) we assumed linkage equilibrium between genetically independent QTL in generation 0, while they simulated linked QTL distributed over four chromosomes of 50 cM, which corresponds to substantial fewer than 200 independent segments. Thus, their comparison of 20 vs. 200 QTL is close to our comparison of 5 vs. 100 QTL,.. Thus, the different outcomes between our simulations and [11] deserves a specific analysis to be deciphered, for example by simulating the organization of selection they retained, while assuming QTL of perfectly known effects and positions, or by including estimation of the marker effects in our model.

We explored a wide range of values for the ratio ${t}_{0}$ of the variance of the gametic standard deviations to the genetic variance, from 0.001 to 0.565. There is little information on the value of this parameter in real populations. Based on results given by Segelke et al. [6], Bijma et al. [10] suggested that the estimated gametic standard deviation varies between $0.32\sqrt{var\left(GEBV\right)}$ and $0.68\sqrt{var\left(GEBV\right)}$. As suggested by [10], this range corresponds roughly to a minimum of $E\left(ECT\right)-3\sqrt{var\left(ECT\right)}$ and a maximum of $\left(ECT\right)+3\sqrt{var\left(ECT\right)}$), giving $6\sqrt{var\left(ECT\right)}= 0.36\sqrt{var\left(GEBV\right)}$, resulting in a ratio ${t}_{0}$ of the order of 0.014.

Cole and VanRaden [1] reported the range of variation of estimates of Mendelian sampling variance for four traits measured in three dairy cattle breeds. We can approximate the value of ${t}_{0}$ on the basis of their Table 1 by assuming that the upper (UBV) and lower (LBV) bounds of the Mendelian sampling variance covers approximately 4 standard deviations, and using that the expected value (EV) of this variance is half of the genetic variance, ${t}_{0}\sim \frac{{\left[\frac{1}{4}(\sqrt{BUV}-\sqrt{BLV})\right]}^{2}}{\frac{1}{4} 2 EV}$. Then, estimates of the ${t}_{0}$ ratio for the four traits evaluated in by [1] range from 0.157 to 0.355 (Table 3), which corresponds to a number of independent QTL of the order of 10. For this number of QTL and with the selection situations that we explored, the discounted cumulative gain surplus over 10 generations would be on average 2.4%, with a maximum of 5.9% (see Additional file 4 Table S2).

**Table 3:** Estimates of the ${t}_{0}$ ratio using results by Cole and VanRaden [1]

Trait	Breed	$${t}_{0}$$
Daughter pregnancy rate (%)	Brown Swiss	0.157
	Holstein	0.269
	Jersey	0.174
Milk yield (kg)	Brown Swiss	0.319
	Holstein	0.297
	Jersey	0.184
Lifetime net merit$ (USD)	Brown Swiss	0.290
	Holstein	0.355
	Jersey	0.279
Protein yield (kg)	Brown Swiss	0.330
	Holstein	0.246
	Jersey	0.306

It is also possible to explore the range of values of ${t}_{0}$ from simulations based on realistic genetic architectures of traits. For this purpose, we used the genotypes (40 K phased SNPs) of 762 Large White boars. Of these, 1 to 200 SNPs were randomly designated as QTL (with effects drawn from a normal distribution), which was replicated 1000 times, For each replicate, the genetic variance was calculated from the QTL genotypes and their effects (i.e. based on the TBV of the 762 boars), and the gametic variances were obtained using the formula of [17]. Results showed values of ${t}_{0}$ ranging from 0.64 to 0.10 when less than 15 QTL were segregating (see Additional file 5 Figure S5), i.e. higher than those estimated from [1] in Table 3. For polygenic traits (beyond 100 QTL), the ${t}_{0}$ ratio was lower than 0.025, and reached 0.014 for 1000 QTL. These values are similar to those presented above (Table 2) for small numbers of QTL and a uniform distribution of allele frequencies, and get higher when more than 100 QTL are simulated, which is due to the non-independence between loci in these last simualions based on a realistic genetic architectures, which reduces the number of independent segments. Thus, it seems that the ratio ${t}_{0}$ takes different values depending on the population and trait. Estimating the value of this parameter is then an essential prerequisite for implementing selection based on UC.

### ***Optimal choice of the weight on gametic variance ***${\varvec{\theta}},$*** in the UC***

In the classical definition of the UC in plant breeding [3–6], the prediction of the gametic variance is weighted by the selection intensity $i(p)$ within F1 families, assuming a selection rate $p$, and UC is the expectation of the $p\_best$ fraction of the lines derived from this F1. In our work, the weight on gametic variance, i.e. $\theta ,$ is a variable to be optimized, the objective being to maximizae the mean genetic value of the population after $T$ generations of selection (or the discounted cumulative gain after 10 generations). We did not found rules for the distribution of $\theta$ optimal values (see Additional file 4 Tables S3 and S4) when varying the number of selected breeders (${S}_{m}$, ${S}_{f}$), the number $Q$ of QTLs, the distribution of allele frequencies (α), and the number of generations $T$, showing that the objective function (${GG}_{T} \; or \; {DCGG}_{10}$) landscape is rather flat around the $\theta =0.5$ value. A better approach would be dynamic optimization of this parameter $\theta ,$ with values changing across the generations but this is highly time consuming. We tested (data not shown) a dynamic programing approach in the very simple case of a trait controlled by two linked QTL. Dynamic optimization of the parameter $\theta$ increased genetic gain in only 10% of the studied situations (defined by the initial alleles frequencies, linkage disequilibrium, recombination rate between the loci, effects of the alleles on the trait, selection rate), the gain being rather limited (less than 2.5%). This is why we did not try to further explore this path.

### Comparison between algebraic approach and simulations

The results (i.e. the relative efficiency of UC compared to classical selection) obtained using the algebraic approach presented in the first part of this paper (Fig. 3) were more optimistic than the results of the simulations (Fig. 7).This is because the algebraic approach was developed in an asymptotic context of an infinite number of genes and individuals, and assumes stability of gametic variance in spite of selection.

When the number of simulated QTL is very large, the distribution of genetic values becomes closer to normality, and the gametic variances no longer change under the effect of selection (Fig. 5). Thus, in the case of 1000 QTL with a uniform allele frequency distribution (${S}_{m}=5$, ${S}_{f}=1000$, $Q=1000$, and $\alpha = 1.0$), resulting in an initial ratio ${t}_{0}$ of 0.001 (Table 2), the algebraic model predicts UC selection to increase genetic gain by 0.63% over classical selection, while 0.82% was obtained with simulations, with the difference not being significant.

To give the algebraic model a broader predictive value, it would be appropriate in the future to remove the assumptions of infinite numbers of reproducers and QTL. Beyond the effects of drift specific to small populations on the variance of Mendelian sampling terms (as for instance described in [18]), one must be able to model the change in the distribution of the gametic variances as a result of selection. And indeed, our results show that this distribution evolves under the effect of selection when the number of independent QTL is limited (Fig. 5).

### Estimation of the SNP effects

The simulations were carried out assuming that the QTL were perfectly located on the genome and had known effects. This assumption allows the relative efficiency of the UC compared to classical selection to be evaluated under the best conditions of application of those selection rules. We note that the additional gain was not significant when the trait was governed by a very large number of independent QTL, and, among all the situations tested, never exceeded 6.6% for very strong allele frequency disparities ($\alpha =0.2$), 3.6% when these frequencies were more balanced ($\alpha \ge 0.8$). In practice, QTL effects must be estimated using data from a genotyped and phenotyped population. QTL estimates are subject to errors and regressed towards zero. As demonstrated for instance by [19], very large families are required (more than 1000 descendants in their case) for the observed gametic standard deviations to be well correlated with their genomic predictions. This may be the case for large dairy cattle populations or fish, but, in other conditions, the efficiency of UC will undoubtedly be greatly reduced, further limiting its attractiveness.

### Extension to mate selection and relation between UC and OCS

Our work focused on optimizing a criterion for ranking breeding candidates. In plant breeding, the objective is also to optimize matings between pure lines using a prediction of the variability of the F1 offspring. A follow-up study for application to animal breeding could also seek to co-optimize the choice of breeders and their matings. In this context, the variance to be considered would no longer be the variance of the gamete values of the candidates, but that of future progeny values of parental pairs candidates, the t-ratio being redefined by comparing this variance to the variance of the average TBV (or GEBV) of the parents. It would be appropriate to compare the effectiveness of this approach (in theory and numerically) to that of existing proposals to optimize matings and the variance of their progeny (e.g. [20–22]).

Selection on UC and Optimal Contribution Selection (OCS) proposed by [23] both consider genetic diversity when selecting individuals for breeding. However, these two methods do this in very different ways. First, these two methods were developed for very different purposes: UC focuses on the trait to be selected and works with genotypes and effects of QTL for the trait, while OCS aims at controlling population inbreeding using co-ancestry coefficients or genomic matrices built with neutral loci as well as QTL. Secondly, the estimation of gametic variances in UC considers the possibility of recombination events during meiosis (e.g. 17), a phenomenon not considered in OCS.

Coming back to the definitions and considering application of OCS based only on QTL genotypes, a major difference between the two methods is that UC selection aims to maximize the expectation of the within family variance, while OCS aims to minimize the between family covariances. If we decompose the genetic variance of a future generation into the sum of the expectation of the within family variance and the variance of the within family expectation, the first term can be managed by UC selection and the second by OCS. With UC selection, the most heterozygous individuals are favoured (their offspring will be the most variable), while OCS aims to produce the most heterozygous progeny. These objectives differ: if we consider a single locus (alleles $A$ and $B$) the best mating with UC is $AB \times AB$, when $AA \times BB$ will be in the top list with OCS.

As proposed by [24] UC selection and OCS can be combined, the choice of the reproducers (which may be defined by vector ${\varvec{c}}$ that assembles $\left\{{c}_{i},i=1\cdots {N}_{m}\right\}$ and $\left\{{c}_{j},j=1\cdots {N}_{f}\right\},$ i.e. the parental contributions for sire *i* and dam *j*, following Meuwissen [23]) being driven by maximization of the mean UC of the selected parents (${{\varvec{c}}}^{\mathbf{^{\prime}}}{\varvec{U}}{\varvec{C}}= \sum_{i}{c}_{i}{UC}_{i}+\sum_{j}{c}_{j}{UC}_{j}$) and minimization of their mean coancestry ${{\varvec{c}}}^{\mathbf{*}\mathbf{^{\prime}}}{\varvec{G}}{{\varvec{c}}}^{\mathbf{*}}$ based on the genomic relationship matrix ${\varvec{G}}$ (${{\varvec{c}}}^{\mathbf{*}}$ being the vector of parental contributions post selection vector, as described in [25]).

In the first generation UC selection and OCS independently influence the genetic variability, but this is probably not true when considering more than one generation. The mean inbreeding (controlled by OCS) is directly linked to the mean heterozygosity of the population, and the gametic variance of a reproducer (controlled by UC) is proportional to its heterozygosity. At the population level, inbreeding coefficients and gametic variances are negatively correlated (e.g. [6]). Thus, when the selection of a generation $G0$ is organised with OCS, generation $G1$ will be more heterozygous, possibly exhausting the efficiency of UC selection applied to this generation, UC selection exploiting variability of hetorozygocity between candidates. On the opposite the generation $G2$ being more variable if UC is used to select the G1 parents. However, it is more difficult to see how applying UC selection in $G0$ can increase the efficiency of OCS in $G1.$ In the future it would be highly useful to optimize the best combination of UC selection and OSC for animal selection, potentially considering dynamic optimization, and compare this optimized combination to simple OCS and simple UC selection.

## Conclusions

For traits controlled by a very large number of QTL with small effects, accounting for the information on the variability of gametic variances provided by genome markers to choose future breeders is of very little interest (in terms of genetic progress generated by recurrent selection). When the effective number of QTL (taking into account linkage disequilibrium, which reduces the number of independent chromosome segments) is small, this information, used in a UC that linearly combines GEBV and an estimate of the gametic standard deviation, can slightly increase long-term genetic gain (with a maximum of 6% in the range of situations studied). As Zhong and Jannink [4] clearly showed, the key factor in deciding whether or not to rank selection candidates on UC rather than on GEBV is the ratio between the variance of the gametic standard deviations and the variance of the GEBV. This ratio depends on the number of QTL and the distribution of their allele frequencies and, thus, these must be estimated as a prerequisite.

## Supplementary Information

Additional file 1: Figure S1. Distribution of allele frequencies according to parameters $\alpha$ and $\beta$.

Additional file 2: Text S1. Effect of selection on UC under the assumption of normality of true breeding values (TBV) and ECT and invariance of mendelian sampling.

Additional file 3: Text S2. Correlation between the numbers of heterozygous QTL in a breeder and its progeny.

Additional file 4: Tables S1–S4. These tables present the comparison of selection on UC and classical selection according to the number of selected breeders (${S}_{m}$, ${S}_{f}$), number $Q$ of QTL and the distribution of allele frequencies (α). Table S1. Relative superiority of selection on UC for the genetic gain after five generations. Table S2. Relative superiority of selection on UC for the cumulative genetic gain after 10 generations. Table S3. Optimal $\theta$ coefficient of the UC for a genetic gain after five generations. Table S4. Optimal $\theta$ coefficient of the UC for a cumulative genetic gain after 10 generations

Additional file 5: Figure S5. Relation between ${t}_{0}$ (the ratio of the variance of the candidate gametic variance and that of half of the candidate genetic values) and the number of QTLs.

## Data Availability

Scripts and programs are available upon reasonable request to the authors.
