# Supplementary material for: Is there an advantage of using genomic information to estimate gametic variances and improve recurrent selection in animal populations?
Source: Genet Sel Evol. 2025 Feb 17;57:5. doi: 10.1186/s12711-025-00953-7 (PMC11831845; doi:10.1186/s12711-025-00953-7)
Supplement: Supplementary file 1 — Additional file 1: Figure S1. Distribution of allele frequencies according to parameters $\alpha$α and $\beta$β. [file 12711_2025_953_MOESM1_ESM.docx]

**Additional file 1 Figure S1**

**Allele frequency distribution**

The frequencies are distributed in a Beta distribution, with parameters$\alpha=\beta$. The shape of this distribution, as well as the average frequency of “rare” alleles ($A_{q}$) are in Figure S1.

**Figure S1: Distribution of allele frequencies according to parameters α and β.**

|  |  |
| --- | --- |
