# Supplementary material for: Is there an advantage of using genomic information to estimate gametic variances and improve recurrent selection in animal populations?
Source: Genet Sel Evol. 2025 Feb 17;57:5. doi: 10.1186/s12711-025-00953-7 (PMC11831845; doi:10.1186/s12711-025-00953-7)
Supplement: Supplementary file 2 — Additional file 2: Text S1. Effect of selection on UC under the assumption of normality of true breeding values (TBV) and ECT and invariance of mendelian sampling. [file 12711_2025_953_MOESM2_ESM.docx]

**Additional file 2 Text S1**

**Effect of selection on UC under the assumption of normality of true breeding values (TBV) and ECT and invariance of mendelian sampling**

We select candidates for reproduction of generation $G_{t}$ based on the criterion ${UC}_{i}=\frac{1}{2}{TBV}_{i}+\theta{ECT}_{i}$, which is the expected value of its gametes. A candidate is selected if its UC is higher that a threshold $s$ . At this generation *the ratio* ${var}_{i}\left( {ECT}_{i} \right)/{var}_{i}\left( \frac{1}{2}{TBV}_{i} \right)$ is noted $t_{t}$. The TBV of the progeny $G_{t+1}$ are assumed distributed in a Gaussian distribution.

Indexing the distribution moments with *i* ($E_{i}$, ${cov}_{i}$, ${var}_{i})$ means that they are obtained on all males *i*. Indexing with *ik* ($E_{ik}$, ${cov}_{ik}$, ${var}_{ik}$) means that they are obtained on all males *i* and their progeny *k*.

At generation $G_{0}$, mendelian samplings are distributed with mean zero and variances ${ECT}_{i0}^{2}=\sigma_{i0}^{2}$, which depend on the male i. We assume with Bijma et al [8], following Bulmer [10], that the distribution of mendelian sampling variances does not change over time, therefore that $E_{i}\left[ \sigma_{it}^{2} \right]=E_{i}\left[ \sigma_{i0}^{2} \right]=c_{\sigma}$ and $E_{i}\left[ \sigma_{it} \right]=E_{i}\left[ \sigma_{i0} \right]=m_{\sigma}$, Thus the variance of the mendelian sampling standard deviations ${var}_{i}\left[ \sigma_{it} \right]={var}_{i}\left[ \sigma_{i0} \right]=E_{i}\left[ \sigma_{i0}^{2} \right]-{E_{i}\left[ \sigma_{i0} \right]}^{2}=c_{\sigma}-m_{\sigma}^{2}=v_{\sigma}$.

The initial ratio is $t_{0}=\frac{v_{\sigma}}{\frac{1}{4}\sigma_{g0}^{2}}$, and, at generation $G_{t}$, it is $t_{t}=\frac{v_{\sigma}}{\frac{1}{4}\sigma_{gt}^{2}}=t_{0}\frac{\sigma_{g0}^{2}}{\sigma_{gt}^{2}}$.

The $H_{ik}$ values of the paternal gametes are $H_{ik}=\frac{1}{2}{TBV}_{i}+A_{ik}$, with $A_{ik}$ the mendelian sampling. Their variance is ${var}_{ik}\left( H_{ik} \right)={var}_{i}\left( \frac{1}{2}{TBV}_{i} \right)+{var}_{ik}\left( A_{ik} \right)$. At generation $G_{0}$ ${var}_{ik}\left( H_{ik} \right)=\frac{1}{2}\sigma_{g0}^{2}$ and ${var}_{i}\left( {TBV}_{i} \right)=\sigma_{g0}^{2}$, therefore ${var}_{ik}\left( A_{ik} \right)=\frac{1}{4}\sigma_{g0}^{2}$. With ${var}_{ik}\left( A_{ik} \right)=E_{i}\left[ {var}_{k}\left( A_{ik} \right) \right]+{var}_{i}\left[ E_{k}\left( A_{ik} \right) \right]=c_{\sigma}+0=c_{\sigma}$, then $c_{\sigma}=\frac{1}{4}\sigma_{g0}^{2}$ and $m_{\sigma}=\frac{1}{2}\sigma_{g0}\sqrt{1-t_{0}}$.

The **mean** of the TBV distribution of progeny $G_{t+1}$ is $\mu_{gt+1}=E_{ik}\left[ {TBV}_{ik}|{UC}_{i}>s \right]=\mu_{gt}+i(p)\frac{{cov}_{ik}({UC}_{i},{TBV}_{ik})}{{var}_{ik}({UC}_{i})}\sqrt{{var}_{ik}({UC}_{i})}$.

To alleviate the notations, the elements of the formulas are not indexed by the generation. Then ${TBV}_{ik}$are the genetic values of the individuals of generation $G_{t+1}$, while ${TBV}_{i}$ $, {UC}_{i}$ and ${ECT}_{i}$ relate to $G_{t}$ breeders.

Assuming ${cov}_{ik}\left( {TBV}_{i},{ECT}_{i} \right)=0$, ${var}_{ik}\left( {UC}_{i} \right)=\frac{1}{4}{var}_{ik}\left( {TBV}_{i} \right)+\theta^{2}{var}_{ik}\left( {ECT}_{i} \right)=\frac{1}{4}\sigma_{gt}^{2}+\theta^{2}v_{\sigma}$.

${cov}_{ik}\left( {UC}_{i},{TBV}_{ik} \right)=\frac{1}{2}{cov}_{ik}\left( {TBV}_{i},{TBV}_{ik} \right)=\frac{1}{4}{var}_{i}\left( {TBV}_{i} \right)=\frac{1}{4}\sigma_{gt}^{2}$, with $v_{\sigma}=\frac{1}{4}\sigma_{gt}^{2}t_{t}$

Then $\mu_{gt+1}=\mu_{gt}+i(p)\frac{\frac{1}{4}\sigma_{gt}^{2}}{\sqrt{\frac{1}{4}\sigma_{gt}^{2}+\theta^{2}v_{\sigma}}}=\mu_{gt}+\frac{1}{2}i(p)\sigma_{gt}\frac{1}{\sqrt{1+\theta^{2}t_{t}}}$

The **variance** of the TBV distribution of progeny $G_{t+1}$ is

$\sigma_{gt+1}^{2}={var}_{ik}\left[ {TBV}_{ik}|{UC}_{i}>s \right]=E_{i}\left[ {var}_{k}\left[ {TBV}_{ik}|i;{UC}_{i}>s \right] \right]+{var}_{i}\left[ E_{k}\left[ {TBV}_{ik}|{i;UC}_{i}>s \right] \right]$

${var}_{k}\left[ {TBV}_{ik}|{i;UC}_{i}>s \right]={ECT}_{i}^{2}+cst$ and $E_{k}\left[ {TBV}_{ik}|{i;UC}_{i}>s \right]=\frac{1}{2}{TBV}_{i}$

The constant (cst) is the part of variance coming from the dams, with value ${\frac{1}{2}\sigma}_{gt}^{2}$.

Therefore, with *s* the selection threshold, ${var}_{ik}\left[ {TBV}_{ik}|{UC}_{i}>s \right]=E_{i}\left[ {ECT}_{i}^{2}|{UC}_{i}>s \right]+{\frac{1}{4}var}_{i}\left[ {TBV}_{i}|{UC}_{i}>s \right]+{\frac{1}{2}\sigma}_{gt}^{2}$

$E_{i}\left[ {ECT}_{i}^{2}|{UC}_{i}>s \right]={E_{i}\left[ {ECT}_{i}|{UC}_{i}>s \right]}^{2}+{var}_{i}\left[ {ECT}_{i}|{UC}_{i}>s \right]$

$E_{i}\left[ {ECT}_{i}|{UC}_{i}>s \right]=m_{\sigma}+i(p)\frac{{cov}_{i}({UC}_{i},{ECT}_{i})}{{var}_{i}({UC}_{i})}\sqrt{{var}_{i}({UC}_{i})}$

$${cov}_{i}({UC}_{i},{ECT}_{i})=\theta{var}_{i}({ECT}_{i})=\theta v_{\sigma}=\theta t_{t}\frac{1}{4}\sigma_{gt}^{2}$$

$${var}_{i}\left( {UC}_{i} \right)=\frac{1}{4}{var}_{i}\left( {TBV}_{i} \right)+\theta^{2}{var}_{i}\left( {ECT}_{i} \right)$$

$$=\frac{1}{4}\sigma_{gt}^{2}+\theta^{2}v_{\sigma}=\frac{1}{4}\sigma_{gt}^{2}(1+\theta^{2}t_{t})$$

$E_{i}\left[ {ECT}_{i}|{UC}_{i}>s \right]=m_{\sigma}+i(p)\frac{\theta v_{\sigma}}{\sqrt{\frac{1}{4}\sigma_{gt}^{2}+\theta^{2}v_{\sigma}}}=m_{\sigma}+i(p)\frac{1}{2}\sigma_{gt}\frac{\theta t_{t}}{\sqrt{1+\theta^{2}t_{t}}}$

${var}_{i}\left[ {ECT}_{i}|{UC}_{i}>s \right]={var}_{i}\left[ {ECT}_{i} \right]\left( 1-\frac{{{cov}_{i}\left( {UC}_{i},{ECT}_{i} \right)}^{2}}{{var}_{i}\left( {UC}_{i} \right){var}_{i}\left( {ECT}_{i} \right)}i\left( p \right)\left( i\left( p \right)-s \right) \right)$ ${var}_{i}\left[ {ECT}_{i}|{UC}_{i}>s \right]=v_{\sigma}\left[ 1-\frac{\left( \theta v_{\sigma} \right)^{2}}{\left( \frac{1}{4}\sigma_{gt}^{2}+\theta^{2}v_{\sigma} \right)v_{\sigma}}i\left( p \right)\left( i\left( p \right)-s \right) \right]$

$=v_{\sigma}\left[ 1-\frac{\left( \theta t_{t}\frac{1}{4}\sigma_{gt}^{2} \right)^{2}}{\left( \frac{1}{4}\sigma_{gt}^{2}(1+\theta^{2}t_{t}) \right)\frac{1}{4}\sigma_{gt}^{2}t_{t}}i(p)\left( i(p)-s \right) \right]$

${var}_{i}\left[ {ECT}_{i}|{UC}_{i}>s \right]=v_{\sigma}\left( 1-\frac{\theta^{2}t_{t}}{1+\theta^{2}t_{t}}i(p)\left( i(p)-s \right) \right)$

$E_{i}\left[ {ECT}_{i}^{2}|{UC}_{i}>s \right]=\left( m_{\sigma}+i(p)\frac{1}{2}\sigma_{gt}\frac{\theta t_{t}}{\sqrt{1+\theta^{2}t_{t}}} \right)^{2}+v_{\sigma}\left( 1-\frac{\theta^{2}t_{t}}{1+\theta^{2}t_{t}}i(p)\left( i(p)-s \right) \right)$

${var}_{i}\left[ {TBV}_{i}|{UC}_{i}>s \right]={var}_{i}\left[ {TBV}_{i} \right](1-\frac{{{cov}_{i}\left( {UC}_{i},{TBV}_{i} \right)}^{2}}{{var}_{i}\left( {UC}_{i} \right){var}_{i}\left( {TBV}_{i} \right)}i(p)\left( i(p)-s \right))$

${var}_{i}\left[ {TBV}_{i}|{UC}_{i}>s \right]=\sigma_{gt}^{2}(1-\frac{{\frac{1}{4}\sigma}_{gt}^{4}}{\left( \frac{1}{4}\sigma_{gt}^{2}+\theta^{2}t_{t}{\frac{1}{4}\sigma}_{gt}^{2} \right)\sigma_{gt}^{2}}i(p)\left( i(p)-s \right))$

${var}_{i}\left[ {TBV}_{i}|{UC}_{i}>s \right]=\sigma_{gt}^{2}(1-\frac{1}{1+\theta^{2}t_{t}}i(p)\left( i(p)-s \right))$

${var}_{ik}\left[ {TBV}_{ik}|{UC}_{i}>s \right]=\left( m_{\sigma}+i(p)\frac{1}{2}\sigma_{gt}\frac{\theta t_{t}}{\sqrt{1+\theta^{2}t_{t}}} \right)^{2}+v_{\sigma}\left( 1-\frac{\theta^{2}t_{t}}{1+\theta^{2}t_{t}}i(p)\left( i(p)-s \right) \right)+\frac{1}{4}\sigma_{gt}^{2}\left( 1-\frac{1}{1+\theta^{2}t_{t}}i\left( p \right)\left( i\left( p \right)-s \right) \right)+{\frac{1}{2}\sigma}_{gt}^{2}$

${var}_{ik}\left[ {TBV}_{ik}|{UC}_{i}>s \right]=\sigma_{gt}^{2}\left( \frac{3}{4}+\frac{1}{4}t_{t} \right)-\sigma_{gt}^{2}i(p)\left( i(p)-s \right)\frac{1}{4}\frac{1+\theta^{2}t_{t}^{2}}{1+\theta^{2}t_{t}}+\left( m_{\sigma}+i(p)\frac{1}{2}\sigma_{gt}\frac{\theta t_{t}}{\sqrt{1+\theta^{2}t_{t}}} \right)^{2}$

When *p*=1, $i(p)$ =0 (no selection) ${var}_{ik}\left[ {TBV}_{ik}|{UC}_{i}>-\infty\right]=\sigma_{g0}^{2}$.
