# Supplementary material for: Is there an advantage of using genomic information to estimate gametic variances and improve recurrent selection in animal populations?
Source: Genet Sel Evol. 2025 Feb 17;57:5. doi: 10.1186/s12711-025-00953-7 (PMC11831845; doi:10.1186/s12711-025-00953-7)
Supplement: Supplementary file 3 — Additional file 3: Text S2. Correlation between the numbers of heterozygous QTL in a breeder and its progeny. [file 12711_2025_953_MOESM3_ESM.docx]

**Additional file 3 Text S2**

**Correlation between the numbers of heterozygous QTL in a breeder and its progeny**

A breeder *i* is characterized by a list of QTL of genotypes $A_{q}B_{q}$, $A_{q}A_{q}$ and $B_{q}B_{q}$. Let $Q_{mi}, Q_{Ai}$ and $Q_{Bi}$ be the lengths of these lists, respectively. A progeny *k* of this breeder will have $xh, xA$ and $xB$ heterozygous QTLs at the loci of these same lists, in total $Q_{mk}$.

We can write that $Q_{mi}=\sum_{q=1}^{Q} S_{qi}$the sum of the heterozygous or non-heterozygous states of each of the $Q$ QTLs in individual *i* ($S_{qi}=1$ if individual *i* is $A_{q}B_{q}$, 0 otherwise). $Q_{mk}$ is defined similarly.

If the QTLs are not linked and in linkage equilibrium $var\left( Q_{mi} \right)=\sum_{q=1}^{Q} var\left( S_{qi} \right)$ and $covar\left( Q_{mi},Q_{mk} \right)=\sum_{q=1}^{Q} \sum_{q'=1}^{Q} covar\left( S_{qi},S_{q'k} \right)=\sum_{q=1}^{Q} covar\left( S_{qi},S_{qk} \right)$.

We assume here that the effects on the trait are the same for all QTLs, these effects will therefore not appear in the formulas.

We first consider the case where the allele frequencies are the same for all QTLs. We note *f* the (shared among QTLs) frequency in the parental generation of alleles $A_{q}, \forall q$, and $fh=F_{A_{q}B_{q}}$.

In this situation, $var\left( Q_{mi} \right)=Qfh(1-fh)$ and $cov\left( Q_{mi},Q_{mk} \right)=Qcov\left( S_{qi},S_{qk} \right)=Q{cov}_{ik}$. With panmixia $fh=2f\left( 1-f \right)$. Then the correlation $r\left( Q_{mi},Q_{mk} \right)$ between $Q_{mi}$ and $Q_{mk}$ does not depend on the number of QTLs: $r\left( Q_{mi},Q_{mk} \right)=\frac{Q{cov}_{ik}}{\sqrt{Qfh(1-fh)Qfh(1-fh)}}=\frac{{cov}_{ik}}{fh(1-fh)}$.

Covariance ${cov}_{ik}=covar\left( S_{qi},S_{qk} \right)=E\left( S_{qi}S_{qk} \right)-E\left( S_{qi} \right)E\left( S_{qk} \right)$

$${cov}_{ik}=prob\left( S_{qk}=1 \right|S_{qi}=1)prob(S_{qi}=1)-prob{(S}_{qk}=1)prob(S_{qi}=1)$$

then ${cov}_{ik}=\frac{1}{2}fh-{fh}^{2}=\frac{1}{2}fh(1-2fh)$.

So $\left( Q_{mi},Q_{mk} \right)=\frac{\frac{1}{2}(1-2fh)}{1-fh}$, and with panmixia $r\left( Q_{mi},Q_{mk} \right)=\frac{1}{2}\frac{1-4f(1-f)}{1-2f(1-f)}$ (Bijma et al [8] reported $\frac{1-4f(1-f)}{1-2f(1-f)}$ as the heritability of heterozygosity, estimated as twice the coefficient of regression $\frac{cov\left( Q_{mi},Q_{mk} \right)}{var\left( Q_{mi} \right)}$).

In the more general case, the allelic frequencies $f_{A_{q}}$ and $f_{B_{q}}$ vary between QTLs. Let $m_{f}^{r}$ be the moment of order *r* of the distribution of $f_{A_{q}}$. Then (always assuming the QTLs are not linked and in linkage equilibrium, so that $F_{A_{q}B_{q}}=2f_{A_{q}}f_{B_{q}}=2f_{A_{q}}\left( 1-f_{A_{q}} \right)={fh}_{q}$), we have $var\left( Q_{mi} \right)=\sum_{q=1}^{Q} var\left( S_{qi} \right)=\sum_{q=1}^{Q} {fh}_{q}(1-{fh}_{q})$ and $covar\left( Q_{mi},Q_{mk} \right)=\sum_{q=1}^{Q} covar\left( S_{qi},S_{qk} \right)=\sum_{q=1}^{Q} \frac{1}{2}{fh}_{q}(1-2{fh}_{q})$. Therefore $r\left( Q_{mi},Q_{mk} \right)=\frac{1}{2}\frac{\sum_{q=1}^{Q} {fh}_{q}\left( 1-2{fh}_{q} \right)}{\sum_{q=1}^{Q} {fh}_{q}\left( 1-{fh}_{q} \right)}$.

If the number of QTL is large enough $\frac{1}{Q}\sum_{q=1}^{Q} {fh}_{q}$ converges to $\left( {fh}_{q} \right)=2m_{f}^{1}-2m_{f}^{2}$ and $\frac{1}{Q}\sum_{q=1}^{Q} {fh}_{q}^{2}$ to $E\left( {fh}_{q}^{2} \right)=4m_{f}^{2}-8m_{f}^{3}+4m_{f}^{4}$. Furthermore assuming that the mean of the ratio is close to the ratio of the means, we can approximate $r\left( Q_{mi},Q_{mk} \right)$ by $\hat{r}\left( Q_{mi},Q_{mk} \right)=\frac{1}{2}\frac{m_{f}^{1}-5m_{f}^{2}+8m_{f}^{3}-4m_{f}^{4}}{m_{f}^{1}-3m_{f}^{2}+4m_{f}^{3}-2m_{f}^{4}}$.

When allele frequencies are distributed in a Beta distribution with parameters α=β, we have

$$m_{f}^{1}=0.5 ; m_{f}^{2}=\frac{\alpha+1}{2(2\alpha+1)} ; m_{f}^{3}=\frac{\alpha+2}{4(2\alpha+1)} ; m_{f}^{4}=\frac{\left( \alpha+2 \right)\left( \alpha+3 \right)}{4(2\alpha+1)(2\alpha+3)}$$
