# Supplementary material for: Is there an advantage of using genomic information to estimate gametic variances and improve recurrent selection in animal populations?
Source: Genet Sel Evol. 2025 Feb 17;57:5. doi: 10.1186/s12711-025-00953-7 (PMC11831845; doi:10.1186/s12711-025-00953-7)
Supplement: Supplementary file 4 — Additional file 4: Tables S1–S4. These tables present the comparison of selection on UC and classical selection according to the number of selected breeders (${S}_{m}$Sm, ${S}_{f}$Sf), number $Q$Q of QTL and the distribution of allele frequencies (α). Table S1. Relative superiority of selection on UC for the genetic gain after five generations. Table S2. Relative superiority of selection on UC for the cumulative genetic gain after 10 generations. Table S3. Optimal $\theta$θ coefficient of the UC for a genetic gain after five generations. Table S4. Optimal $\theta$θ coefficient of the UC for a cumulative genetic gain after 10 generations [file 12711_2025_953_MOESM4_ESM.docx]

**Additional file 4 Tables S1, S2, S3 and S4**

**Comparison of selection on UC and classic selection according to the number of selected breeders (**$\boldsymbol{S}_{\boldsymbol{m}}$**,** $\boldsymbol{S}_{\boldsymbol{f}}$**), number** $\boldsymbol{Q}$ **of QTLs and the distribution of allele frequencies (α)**

**Table S1:** **Relative superiority of selection on UC for the genetic gain after 5 generations**

| $S_{m}$ | $S_{f}$ | $Q$ | $\alpha=0.2$ | $\alpha=0.4$ | $\alpha=0.6$ | $\alpha=0.8$ | $\alpha=1.0$ |
| --- | --- | --- | --- | --- | --- | --- | --- |
| 5 | 1000 | 5 | 5.0% | 3.2% | 1.6% | 1.7% | 0.5% |
| 10 | 1000 | 5 | 2.3% | 2.1% | 0.6% | 0.8% | 0.6% |
| 50 | 1000 | 5 | 0.0% | 2.8% | 2.8% | 0.0% | 0.0% |
| 5 | 500 | 5 | 2.3% | 2.0% | 1.4% | 1.1% | 0.4% |
| 10 | 500 | 5 | 4.0% | 2.8% | 0.9% | 0.6% | 0.5% |
| 50 | 500 | 5 | 2.9% | 1.7% | 1.4% | 0.6% | 0.6% |
| 5 | 50 | 5 | 1.8% | 1.8% | 5.1% | 2.1% | 1.4% |
| 10 | 50 | 5 | 3.3% | 2.9% | 2.7% | 2.4% | 1.3% |
| 50 | 50 | 5 | 2.4% | 1.1% | 1.2% | 0.5% | 0.5% |
| 5 | 1000 | 10 | 4.4% | 4.1% | 3.0% | 1.9% | 1.3% |
| 10 | 1000 | 10 | 5.1% | 3.5% | 2.5% | 1.9% | 1.1% |
| 50 | 1000 | 10 | 0.0% | 1.4% | 0.0% | 1.3% | 0.0% |
| 5 | 500 | 10 | 4.9% | 3.8% | 2.9% | 2.4% | 1.2% |
| 10 | 500 | 10 | 4.9% | 4.6% | 3.3% | 2.0% | 1.7% |
| 50 | 500 | 10 | 2.8% | 1.2% | 1.3% | 1.4% | 0.8% |
| 5 | 50 | 10 | 6.5% | 4.1% | 4.6% | 3.0% | 3.5% |
| 10 | 50 | 10 | 7.5% | 5.5% | 5.0% | 3.8% | 4.2% |
| 50 | 50 | 10 | 4.7% | 3.7% | 2.9% | 1.2% | 1.6% |
| 5 | 1000 | 100 | 5.2% | 3.0% | 2.8% | 2.2% | 2.0% |
| 10 | 1000 | 100 | 3.0% | 2.4% | 2.8% | 2.3% | 1.4% |
| 50 | 1000 | 100 | 1.4% | 1.6% | 1.6% | 1.5% | 1.3% |
| 5 | 500 | 100 | 5.6% | 3.9% | 3.7% | 3.1% | 2.6% |
| 10 | 500 | 100 | 4.6% | 2.8% | 2.8% | 2.1% | 1.8% |
| 50 | 500 | 100 | 2.3% | 2.1% | 1.0% | 1.3% | 0.7% |
| 5 | 50 | 100 | 5.7% | 4.7% | 3.9% | 3.8% | 4.4% |
| 10 | 50 | 100 | 7.9% | 5.9% | 6.0% | 3.8% | 3.9% |
| 50 | 50 | 100 | 5.4% | 4.4% | 3.0% | 2.6% | 1.9% |
| 5 | 1000 | 1000 | 1.4% | 1.8% | 0.4% | 1.3% | 1.0% |
| 10 | 1000 | 1000 | 0.3% | 0.4% | 1.4% | 1.0% | 0.0% |
| 50 | 1000 | 1000 | 0.9% | 0.7% | 0.0% | 1.1% | 1.3% |
| 5 | 500 | 1000 | 3.3% | 2.3% | 1.5% | 1.4% | 1.1% |
| 10 | 500 | 1000 | 2.7% | 2.2% | 1.6% | 1.3% | 0.8% |
| 50 | 500 | 1000 | 1.3% | 1.8% | 0.9% | 0.8% | 1.0% |
| 5 | 50 | 1000 | 4.1% | 2.7% | 2.1% | 1.9% | 1.6% |
| 10 | 50 | 1000 | 4.8% | 3.4% | 2.1% | 2.5% | 2.6% |
| 50 | 50 | 1000 | 4.4% | 2.9% | 2.1% | 1.5% | 1.4% |

**Table S2:** **Relative superiority of selection on UC for the cumulative genetic gain after 10 generations**

| $S_{m}$ | $S_{f}$ | $Q$ | $\alpha=0.2$ | $\alpha=0.4$ | $\alpha=0.6$ | $\alpha=0.8$ | $\alpha=1.0$ |
| --- | --- | --- | --- | --- | --- | --- | --- |
| 5 | 1000 | 5 | 4.2% | 2.6% | 1.1% | 1.3% | 0.3% |
| 10 | 1000 | 5 | 1.9% | 1.9% | 0.4% | 0.6% | 0.2% |
| 50 | 1000 | 5 | 0.0% | 3.1% | 3.1% | 0.2% | 0.1% |
| 5 | 500 | 5 | 1.4% | 1.2% | 1.0% | 0.7% | 0.3% |
| 10 | 500 | 5 | 3.0% | 2.4% | 0.6% | 0.1% | 0.1% |
| 50 | 500 | 5 | 2.9% | 1.0% | 1.5% | 0.2% | 0.6% |
| 5 | 50 | 5 | 1.5% | 1.2% | 4.7% | 1.5% | 1.1% |
| 10 | 50 | 5 | 2.8% | 2.5% | 2.2% | 2.0% | 1.1% |
| 50 | 50 | 5 | 1.7% | 1.1% | 1.1% | 0.2% | 0.3% |
| 5 | 1000 | 10 | 3.9% | 3.8% | 2.6% | 1.2% | 1.0% |
| 10 | 1000 | 10 | 5.1% | 3.4% | 2.5% | 1.7% | 1.1% |
| 50 | 1000 | 10 | 1.4% | 2.7% | 0.0% | 1.3% | 0.2% |
| 5 | 500 | 10 | 4.2% | 3.0% | 2.2% | 1.5% | 0.9% |
| 10 | 500 | 10 | 3.8% | 3.6% | 2.7% | 1.5% | 1.3% |
| 50 | 500 | 10 | 3.1% | 1.3% | 1.3% | 1.2% | 0.7% |
| 5 | 50 | 10 | 5.7% | 3.3% | 3.9% | 2.3% | 2.9% |
| 10 | 50 | 10 | 6.6% | 4.5% | 4.1% | 3.2% | 3.6% |
| 50 | 50 | 10 | 3.8% | 3.0% | 2.3% | 0.9% | 1.2% |
| 5 | 1000 | 100 | 3.4% | 2.7% | 1.6% | 1.9% | 1.7% |
| 10 | 1000 | 100 | 2.2% | 1.3% | 1.7% | 1.3% | 0.9% |
| 50 | 1000 | 100 | 0.9% | 0.4% | 0.1% | 1.1% | 0.4% |
| 5 | 500 | 100 | 5.4% | 3.4% | 2.8% | 2.6% | 2.5% |
| 10 | 500 | 100 | 4.1% | 3.5% | 2.4% | 2.0% | 1.7% |
| 50 | 500 | 100 | 2.3% | 2.4% | 1.6% | 1.2% | 1.2% |
| 5 | 50 | 100 | 3.5% | 2.4% | 1.8% | 1.7% | 1.5% |
| 10 | 50 | 100 | 4.2% | 3.2% | 1.9% | 2.4% | 2.8% |
| 50 | 50 | 100 | 5.9% | 3.4% | 3.0% | 2.3% | 2.2% |
| 5 | 1000 | 1000 | 1.6% | 0.7% | 0.8% | 1.4% | 0.5% |
| 10 | 1000 | 1000 | 0.2% | 1.0% | 0.7% | 0.3% | 0.1% |
| 50 | 1000 | 1000 | 0.0% | 0.5% | 1.0% | 0.9% | 1.3% |
| 5 | 500 | 1000 | 1.9% | 0.5% | 1.5% | 1.9% | 1.1% |
| 10 | 500 | 1000 | 1.0% | 0.9% | 0.5% | 0.9% | 0.2% |
| 50 | 500 | 1000 | 0.6% | 0.7% | 0.9% | 0.9% | 0.2% |
| 5 | 50 | 1000 | 0.3% | 0.8% | 1.7% | 0.9% | 0.4% |
| 10 | 50 | 1000 | 1.7% | 2.0% | 2.7% | 1.5% | 1.6% |
| 50 | 50 | 1000 | 1.8% | 1.4% | 1.3% | 0.9% | 1.3% |

**Table S3:** **Optimal θ coefficient of the UC for a genetic gain after 5 generations**

| $S_{m}$ | $S_{f}$ | $Q$ | $\alpha=0.2$ | $\alpha=0.4$ | $\alpha=0.6$ | $\alpha=0.8$ | $\alpha=1.0$ |
| --- | --- | --- | --- | --- | --- | --- | --- |
| 5 | 1000 | 5 | 0.5 | 0.5 | 0.5 | 0.5 | 0.5 |
| 10 | 1000 | 5 | 0.5 | 0.2 | 0.4 | 0.3 | 0.4 |
| 50 | 1000 | 5 | 0.4 | 0.4 | 0.2 | 0.5 | 0.7 |
| 5 | 500 | 5 | 0.5 | 0.5 | 0.5 | 0.5 | 0.4 |
| 10 | 500 | 5 | 0.5 | 0.4 | 0.4 | 0.5 | 0.5 |
| 50 | 500 | 5 | 0.4 | 0.5 | 0.2 | 0.4 | 0.4 |
| 5 | 50 | 5 | 0.5 | 0.5 | 0.4 | 0.5 | 0.4 |
| 10 | 50 | 5 | 0.5 | 0.3 | 0.4 | 0.5 | 0.5 |
| 50 | 50 | 5 | 0.5 | 0.5 | 0.5 | 0.5 | 0.5 |
| 5 | 1000 | 10 | 0.4 | 0.5 | 0.5 | 0.5 | 0.5 |
| 10 | 1000 | 10 | 0.4 | 0.5 | 0.3 | 0.5 | 0.2 |
| 50 | 1000 | 10 | 1.1 | 0.4 | 0.2 | 0.3 | 0.2 |
| 5 | 500 | 10 | 0.5 | 0.5 | 0.5 | 0.5 | 0.4 |
| 10 | 500 | 10 | 0.5 | 0.5 | 0.5 | 0.5 | 0.5 |
| 50 | 500 | 10 | 0.3 | 0.4 | 0.5 | 0.3 | 0.3 |
| 5 | 50 | 10 | 0.5 | 0.5 | 0.5 | 0.5 | 0.5 |
| 10 | 50 | 10 | 0.5 | 0.5 | 0.5 | 0.5 | 0.5 |
| 50 | 50 | 10 | 0.5 | 0.5 | 0.5 | 0.5 | 0.5 |
| 5 | 1000 | 100 | 0.4 | 0.5 | 0.4 | 0.5 | 0.7 |
| 10 | 1000 | 100 | 0.3 | 0.6 | 0.4 | 0.2 | 0.5 |
| 50 | 1000 | 100 | 0.3 | 0.2 | 0.4 | 0.2 | 0.2 |
| 5 | 500 | 100 | 0.5 | 0.8 | 0.6 | 0.7 | 0.8 |
| 10 | 500 | 100 | 0.7 | 0.7 | 0.6 | 0.3 | 0.4 |
| 50 | 500 | 100 | 0.3 | 0.3 | 0.6 | 0.2 | 0.5 |
| 5 | 50 | 100 | 0.5 | 0.5 | 0.5 | 0.5 | 0.5 |
| 10 | 50 | 100 | 0.5 | 0.5 | 0.5 | 0.6 | 0.4 |
| 50 | 50 | 100 | 0.6 | 0.7 | 0.7 | 1 | 0.7 |
| 5 | 1000 | 1000 | 0.5 | 1 | 0.7 | 0.7 | 0.2 |
| 10 | 1000 | 1000 | 0.6 | 0.5 | 0.4 | 0.9 | 0.4 |
| 50 | 1000 | 1000 | 0.4 | 0.4 | 0.4 | 0.5 | 0.5 |
| 5 | 500 | 1000 | 0.6 | 0.9 | 0.8 | 0.4 | 0.9 |
| 10 | 500 | 1000 | 0.3 | 0.2 | 0.4 | 0.4 | 0.8 |
| 50 | 500 | 1000 | 0.7 | 0.3 | 0.4 | 0.8 | 0.4 |
| 5 | 50 | 1000 | 0.5 | 0.7 | 0.4 | 0.8 | 0.6 |
| 10 | 50 | 1000 | 0.9 | 0.8 | 0.6 | 0.9 | 1 |
| 50 | 50 | 1000 | 1 | 0.8 | 0.8 | 0.6 | 0.9 |

**Table S4:** **Optimal θ coefficient of the UC for a cumulative genetic gain after 10 generations**

| $S_{m}$ | $S_{f}$ | $Q$ | $\alpha=0.2$ | $\alpha=0.4$ | $\alpha=0.6$ | $\alpha=0.8$ | $\alpha=1.0$ |
| --- | --- | --- | --- | --- | --- | --- | --- |
| 5 | 1000 | 5 | 0.4 | 0.5 | 0.5 | 0.5 | 0.5 |
| 10 | 1000 | 5 | 0.5 | 0.2 | 0.3 | 0.3 | 0.4 |
| 50 | 1000 | 5 | 1.1 | 0.4 | 0.2 | 0.2 | 0.2 |
| 5 | 500 | 5 | 0.4 | 0.5 | 0.4 | 0.5 | 0.3 |
| 10 | 500 | 5 | 0.5 | 0.4 | 0.3 | 0.5 | 0.2 |
| 50 | 500 | 5 | 0.3 | 0.5 | 0.2 | 0.3 | 0.2 |
| 5 | 50 | 5 | 0.4 | 0.5 | 0.4 | 0.5 | 0.4 |
| 10 | 50 | 5 | 0.5 | 0.3 | 0.4 | 0.5 | 0.5 |
| 50 | 50 | 5 | 0.5 | 0.4 | 0.4 | 0.5 | 0.5 |
| 5 | 1000 | 10 | 0.4 | 0.5 | 0.5 | 0.5 | 0.5 |
| 10 | 1000 | 10 | 0.4 | 0.5 | 0.3 | 0.5 | 0.2 |
| 50 | 1000 | 10 | 0.4 | 0.4 | 0.4 | 0.3 | 0.3 |
| 5 | 500 | 10 | 0.5 | 0.5 | 0.5 | 0.5 | 0.4 |
| 10 | 500 | 10 | 0.5 | 0.5 | 0.5 | 0.5 | 0.5 |
| 50 | 500 | 10 | 0.3 | 0.4 | 0.5 | 0.3 | 0.3 |
| 5 | 50 | 10 | 0.5 | 0.5 | 0.5 | 0.5 | 0.5 |
| 10 | 50 | 10 | 0.5 | 0.5 | 0.5 | 0.4 | 0.5 |
| 50 | 50 | 10 | 0.5 | 0.5 | 0.5 | 0.4 | 0.5 |
| 5 | 1000 | 100 | 0.6 | 0.5 | 0.4 | 0.5 | 0.7 |
| 10 | 1000 | 100 | 0.6 | 0.6 | 0.6 | 0.5 | 0.9 |
| 50 | 1000 | 100 | 0.3 | 0.5 | 0.2 | 0.2 | 0.2 |
| 5 | 500 | 100 | 0.6 | 0.8 | 0.6 | 0.8 | 0.8 |
| 10 | 500 | 100 | 0.6 | 0.7 | 0.6 | 0.8 | 0.7 |
| 50 | 500 | 100 | 0.7 | 0.6 | 0.6 | 0.6 | 0.5 |
| 5 | 50 | 100 | 0.5 | 0.5 | 0.5 | 0.5 | 0.5 |
| 10 | 50 | 100 | 0.5 | 0.5 | 0.5 | 0.6 | 0.5 |
| 50 | 50 | 100 | 0.6 | 0.7 | 0.7 | 0.8 | 0.7 |
| 5 | 1000 | 1000 | 0.8 | 0.9 | 0.7 | 0.8 | 0.9 |
| 10 | 1000 | 1000 | 1 | 0.4 | 0.4 | 0.9 | 0.4 |
| 50 | 1000 | 1000 | 1 | 0.9 | 0.5 | 0.7 | 0.2 |
| 5 | 500 | 1000 | 0.8 | 1 | 0.8 | 0.8 | 0.7 |
| 10 | 500 | 1000 | 0.7 | 0.9 | 0.6 | 0.9 | 0.7 |
| 50 | 500 | 1000 | 0.7 | 0.8 | 0.4 | 0.8 | 0.4 |
| 5 | 50 | 1000 | 0.5 | 0.7 | 0.4 | 0.6 | 0.6 |
| 10 | 50 | 1000 | 0.9 | 1 | 0.6 | 0.9 | 1 |
| 50 | 50 | 1000 | 1 | 0.8 | 0.9 | 0.6 | 0.9 |
