# Supplementary material for: Is there an advantage of using genomic information to estimate gametic variances and improve recurrent selection in animal populations?
Source: Genet Sel Evol. 2025 Feb 17;57:5. doi: 10.1186/s12711-025-00953-7 (PMC11831845; doi:10.1186/s12711-025-00953-7)

Additional file 5 : Simulation of the $\boldsymbol{t}_{\mathbf{0}}$ ratio

We used the genotypes (40K phased SNPs) of 762 Large White boars. By repeating the draws 1000 times, 1 to 200 SNPs were randomly designated as QTL (with effects drawn from a normal distribution). For each repetition, the genetic variance was calculated from the QTL genotypes and their effects (therefore the TBV of the 762 boars), and the gametic variances were obtained using the formula of [16].

**Figure S5: Estimation by simulation of the** $\boldsymbol{t}_{\boldsymbol{0}}$ **ratio depending on the number of QTL (base data: 762 phased Large White pig genomes).**


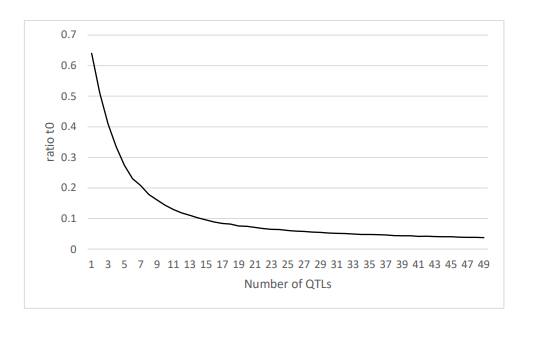

Supplement: Supplementary file 5 — Additional file 5: Figure S5. Relation between ${t}_{0}$t0 (the ratio of the variance of the candidate gametic variance and that of half of the candidate genetic values) and the number of QTLs. [file 12711_2025_953_MOESM5_ESM.docx]
